# A MXene-Based Bionic Cascaded-Enzyme Nanoreactor for Tumor Phototherapy/Enzyme Dynamic Therapy and Hypoxia-Activated Chemotherapy

**DOI:** 10.1007/s40820-021-00761-w

**Published:** 2021-12-09

**Authors:** Xiaoge Zhang, Lili Cheng, Yao Lu, Junjie Tang, Qijun Lv, Xiaomei Chen, You Chen, Jie Liu

**Affiliations:** 1 School of Biomedical Engineering, Shenzhen Campus of Sun Yat-Sen University, No. 66, Gongchang Road, Guangming District, Shenzhen, Guangdong 518107 People’s Republic of China; 2 Department of General Surgery, The Third Affiliated Hospital of Sun Yat-Sen University, Guangzhou, Guangdong 510006 People’s Republic of China

**Keywords:** Cascaded-enzyme nanoreactor, Deoxygenation-sensitive prodrugs, Tumor enzyme dynamic therapy, Phototherapy, CD47

## Abstract

The enzyme-mediated elevation of reactive oxygen species (ROS) at the tumor sites has become an emerging strategy for regulating intracellular redox status for anticancer treatment. Herein, we proposed a camouflaged bionic cascaded-enzyme nanoreactor based on Ti_3_C_2_ nanosheets for combined tumor enzyme dynamic therapy (EDT), phototherapy and deoxygenation-activated chemotherapy. Briefly, glucose oxidase (GOX) and chloroperoxidase (CPO) were chemically conjugated onto Ti_3_C_2_ nanosheets, where the deoxygenation-activated drug tirapazamine (TPZ) was also loaded, and the Ti_3_C_2_-GOX-CPO/TPZ (TGCT) was embedded into nanosized cancer cell-derived membrane vesicles with high-expressed CD47 (m_e_TGCT). Due to biomimetic membrane camouflage and CD47 overexpression, m_e_TGCT exhibited superior immune escape and homologous targeting capacities, which could effectively enhance the tumor preferential targeting and internalization. Once internalized into tumor cells, the cascade reaction of GOX and CPO could generate HClO for efficient EDT. Simultaneously, additional laser irradiation could accelerate the enzymic-catalytic reaction rate and increase the generation of singlet oxygen (^1^O_2_). Furthermore, local hypoxia environment with the oxygen depletion by EDT would activate deoxygenation-sensitive prodrug for additional chemotherapy. Consequently, m_e_TGCT exhibits amplified synergistic therapeutic effects of tumor phototherapy, EDT and chemotherapy for efficient tumor inhibition. This intelligent cascaded-enzyme nanoreactor provides a promising approach to achieve concurrent and significant antitumor therapy.

**Supplementary Information:**

The online version contains supplementary material available at 10.1007/s40820-021-00761-w.

## Highlights

- Gene-engineering tumor cell membrane with CD47 over-expression is achieved to improve the macrophage-mediated phagocytosis of tumor cells by blocking CD47 immune checkpoint.
- A cascade-enzyme nanoreactor combining tumor enzyme dynamic therapy, phototherapy, and deoxygenation-activated chemotherapy is proposed.
- Glucose oxidase and chloroperoxidase can generate sufficient HClO to kill normoxic tumor cells, and tirapazamine can be subsequently activated to kill hypoxic tumor cells.

**Supplementary Information:**

The online version contains supplementary material available at 10.1007/s40820-021-00761-w.

## Graphical abstract

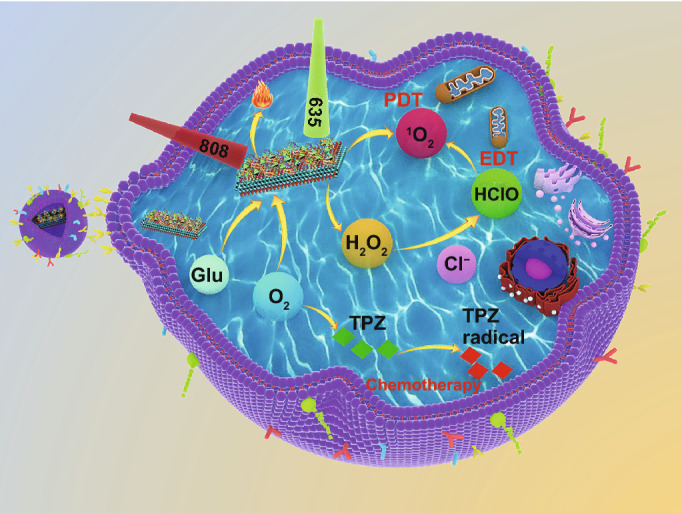

## Introduction

Malignant tumors are still major diseases that threaten human health, and the number of cancer death has increased by nearly 20% in the past 10 years [1]. Among kinds of conventional clinical cancer therapies, chemotherapy is still widely used in clinic as the main treatment mean except for surgery, but the toxic side effects caused by drug resistance, high invasiveness and lack of specific targeting significantly limit its therapeutic effect [2–4]. Therefore, it is urgent to study advanced treatments of cancer. In most recent study, a new kind of strategy so called enzyme dynamic therapy (EDT) provided an innovative approach for tumor treatment by fully taking advantage of the enzyme-catalyzed reactions in tumor microenvironment (TME) to generate different kinds of cytotoxic reactive oxygen species (ROS) [5, 6]. Proverbially, ROS is responsible for regulating intracellular signaling pathways and redox environments [7–9]. The redox environment of tumor cells has better selectivity than normal cells, and rapid accumulation of ROS in a short time can break the vulnerable redox balance and increase the oxidative stress levels in the cells, eventually leading to cancer cell damage and death [10–13]. Therefore, the ROS elevation is considered as an effective way to improve the effect of tumor treatment .

Among the ROS family, highly reactive hypochlorous acid (HClO) is the strongest factor, which can be produced by chloroperoxidase (CPO) catalysis to increase intracellular oxidative stress level and irreversibly destroy proteins, nucleic acids, lipids and carbohydrates [14]. As a highly active peroxidase of calcium-rich yeast with strong antioxidant inactivation ability, CPO enzyme can catalyze the reaction of chlorine ion (Cl^−^) and hydrogen peroxide (H_2_O_2_) to generate HClO and the subsequent decomposition of HClO can produce singlet oxygen (^1^O_2_), forming the CPO-H_2_O_2_-Halogen system for antitumor effect and killing microorganisms [15–17]. However, CPO-mediated catalytic reaction alone is inefficient for HClO generation owing to the low level of H_2_O_2_ in the tumor region [18]. Therefore, it is necessary to elevate the level of H_2_O_2_ in the tumor region for the improvement of the HClO-mediated antitumor effect. So far, various kinds of strategies have been designed to elevate the endogenous H_2_O_2_, such as glucose oxidase (GOX) biocatalysis, nicotinamide adenine dinucleotide phosphate (NADPH) oxidase biocatalysis and superoxide dismutase (SOD) biocatalysis [5, 17, 19]. There into, GOX has been applied repeatedly as an H_2_O_2_ generator for its particular enzyme activity of catalyzing glucose into abundant H_2_O_2_ and gluconic acid, and this process can also consume tumor cell energy to achieve tumor starvation and death [20–23].

In this work, GOX and CPO were designed as the cascaded-enzyme reactor to cut off the intratumoral glucose supply and generate sustained HClO for effective tumor starvation treatment and EDT without external energy. However, the O_2_ consumption in the tumor tissue during starvation treatment exacerbates the degree of hypoxia in the TME, which in turn limits the effectiveness of tumor treatment and induces tumor recurrence and metastasis [24, 25]. To kill the hypoxic tumor cell, tirapazamine (TPZ), as an deoxygenation-sensitive prodrug, can be converted to cytotoxic instantaneous intermediates (Benzotriazine, BTZ) that can induce double-stranded DNA rupture and the hypoxic cells death by topoisomerase II-dependence process [26, 27]. Therefore, the combination of the cascaded-enzyme and TPZ may achieve amplified antitumor therapeutic effects. Besides, to protect enzymes from protein structure degradation and deactivation and deliver multiple components with high drug loading for effectively EDT and chemotherapy simultaneously, two-dimensional transition metal carbides/nitrides (2D MXenes) with good biocompatibility and high specific surface area [28, 29] were applied to co-load the cascaded-enzyme and drug to improve the accumulation in the tumor sites. In the meantime, 2D MXenes can transfer external energy for the generation of exogenous ROS to effectively cause tumor cell apoptosis under laser irradiation [30–32]. Therefore, a strategy of combining physical and biological enzymatic reactions for the enhanced antitumor effect is constructed, for which the laser irradiation can provide short-term exogenous ROS and the enzyme can provide sustainable endogenous ROS.

**Scheme 1:**
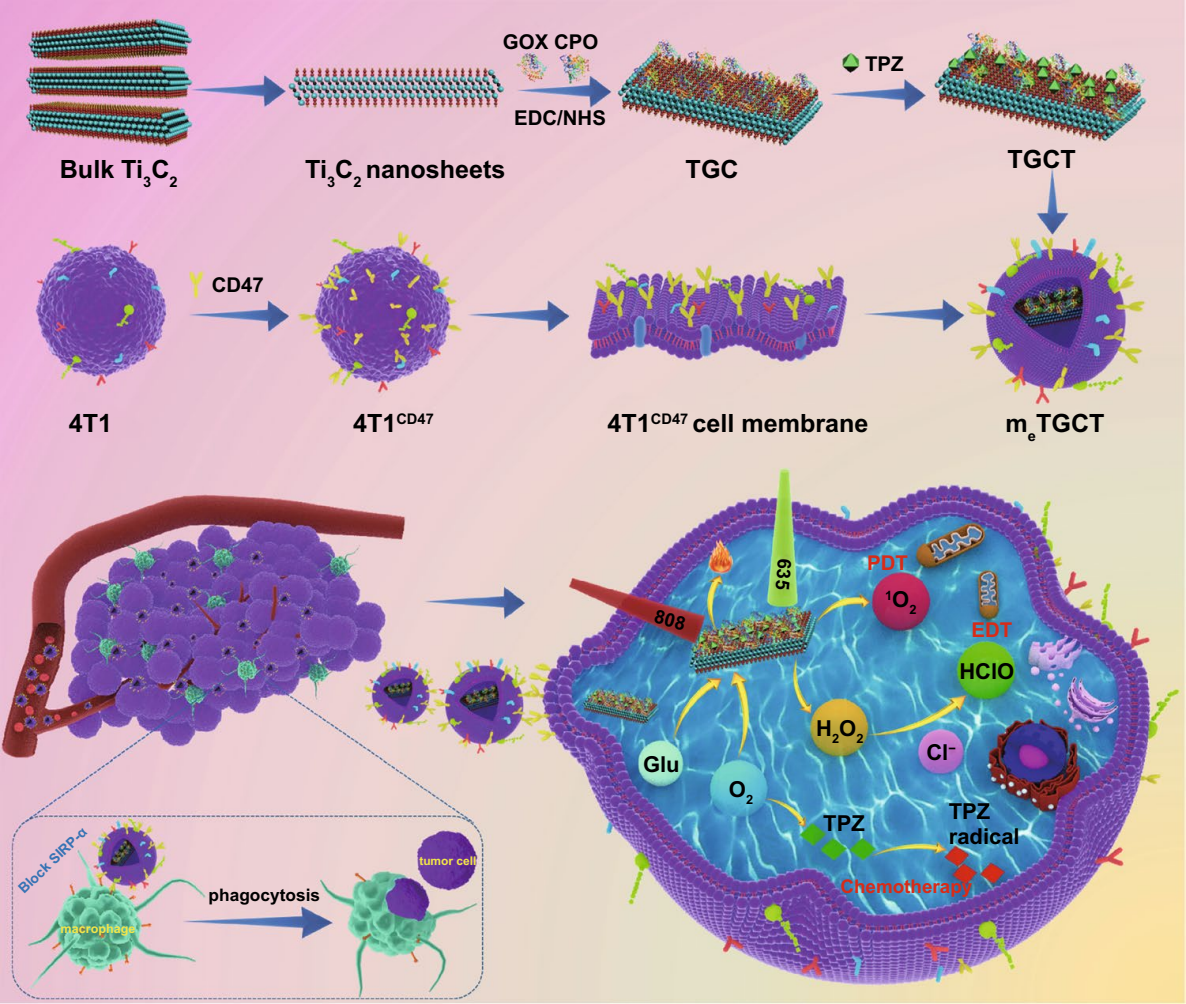
Schematic diagram of the construction of bionic cascaded-enzyme nanoreactor and proposed mechanism in vivo

Herein, we designed and proposed a camouflaged nano-cascaded catalytic system by combining tumor enzyme dynamic therapy, phototherapy and hypoxic-activated chemotherapy for efficient antitumor effects (Scheme 1). In brief, the nano-cascaded enzymes Ti_3_C_2_-GOX-CPO/TPZ (TGCT) were prepared by conjugating GOX and CPO onto the Ti_3_C_2_ 2D nanosheets with TPZ drug loading, then CD47-overexpressed cancer cell membrane biomimetic modification was utilized to form a bionic cascaded-enzyme nanoreactor (denoted as m_e_TGCT). As CD47 can combine with the signal regulatory protein α (SIRPα) to form an effective “don’t eat me” signal, the m_e_TGCT can avoid being eliminated by the innate immune system and block the phagocytosis by the first responder cells (such as macrophages) to realize long blood circulation in vivo [33, 34]. At the same time, because of superior immune escape and homologous targeting capacities, m_e_TGCT would preferentially accumulate in the tumor sites and target tumor cells, which could reduce side effects. Moreover, when m_e_TGCT arrived at the tumor site, high-expressed CD47 could also block the SIRPɑ receptor of the macrophage, thus enhancing the phagocytosis of tumor cells. On the other hand, once m_e_TGCT is internalized by tumor cells, the cascaded-enzyme consumes the glucose and O_2_ in tumor regions to generate the cytotoxic HClO. Simultaneously, Ti_3_C_2_ can produce heat and ROS under NIR laser irradiation, and heat can accelerate the enzyme-catalyzed reaction rate and ROS production, further aggravating the hypoxic status in TME. Then, TPZ as a hypoxia-activated prodrug can be activated by reductase and further cause double-stranded DNA rupture and cell apoptosis to enhance the effects of EDT and phototherapy. Consequently, this camouflaged cascaded-enzyme nanoreactor can realize amplified synergistic effects of the phototherapy/enzyme dynamic therapy and hypoxia-activated chemotherapy to inhibit tumor growth efficiently.

## Experimental Methods

### Materials and Instruments

#### Materials

The transfection CD47-expression vector was obtained from Vector Builder Inc. (Guangzhou). Ti_3_C_2_ crystals were bought from Hangzhou Nano Technology Co. Ltd. Carbodiimide hydrochloride (EDC·HCl), N-Hydroxysuccinimide (NHS) and lipopolysaccharide (LPS) were from Sigma-Aldrich. Thiazolyl Blue Tetrazolium Bromide (MTT), Cyanine5.5 amine (Cy5.5-NH_2_) and diphenylisobenzofuran (DPBF) were from Aladdin Reagents. Calcein-AM/PI and Annexin V-FITC/PI Detection Kit were bought from Key GEN Bio TECH (Shanghai, China). Phenylmethanesulfonyl fluoride (PMSF), SDS polyacrylamide gel, chemiluminescent kit, bovine serum albumin (BSA), BCA Protein Assay Kit were bought from the Beytotime Institute of Biotechnology (Guangzhou). Polyvinylidene fluoride (PVDF) membrane was from Millipore Ltd (Shanghai). The anti-CD47, anti-GAPDH and the second antibody were bought from Solarbio life Science (Guangzhou). 2′, 7′-dichlorofluorescein diacetate (DCFH-DA) and 4′, 6-diamidino-2-phenylindole (DAPI) were obtained from Biosharp. Fetal bovine serum and DMEM medium were acquired from Gibco Life Technologies. Anti-CD47/PE antibody was purchased from Bio Legend, Inc. (San Diego, USA). Glucose oxidase (GOX, 1 g), Chloroperoxidase (CPO, 12.5 KU mL^−1^) and Coomassie blue were purchased from Sigma-Aldrich. The Amplite® Colorimetric Hypochlorite (Hypochlorous Acid) Assay Kit was from AAT Bioquest (USA). Hypoxyprobe™-1 plus kit was from Beijing Biolead Biology SCI & TECH Co. Ltd (Beijing), methylene blue (MB), coumarin-6 (C6) and Tirapazamine (TPZ) were from Aladdin Reagents. Mouse mammary tumor cells (4T1) and macrophages (RAW264.7) were from the cell bank of Chinese Academy of Sciences (Shanghai).

#### Instruments

The particle size and zeta potential were measured by Nano zetasizer (Malven). The morphologies were observed using transmission electron microscope (TEM, H7650, Japan) and field emission scanning electron microscope (CFSEM, ZEISS GeminiSEM500, Germany). The crystal structure was measured by X-ray diffraction (XRD, Bruker Quantax Flat Quad 5060, Germany). The Avanti mini extruder was obtained from Avanti Polar Lipids. The UV spectra were obtained by a spectrophotometer (DU-730, USA). Real-time detection of O_2_ concentration was measured by a dissolved O_2_ meter (JPBJ-608, Rex). The cell fluorescence images were performed using a Confocal laser scanning microscope (CLSM, FV3000, Olympus). The cell fluorescence quantitative assay was carried out using flow cytometry (FACSCalibur, BD). The cell viability was assessed with a microplate reader (Bio-Rad, model 550, USA). The temperature change was measured by an infrared thermal imager (Ti27, Fluke). The fluorescence imaging experiments in vivo were performed via IVIS Lumina Vivo Imaging System (PerkinElmer). The tissue sections were detected by Motic AE31 optical microscope (Xiamen).

### Synthesis of m_e_TGCT (CD47-Engineering Membrane@Ti_3_C_2_-GOX-CPO/TPZ)

#### Extraction and Characterization of 4T1 Cell Membrane

4T1 cells with high expression of CD47 were prepared according to our previous study by transfection using lentiviruses plasmid [35]. Subsequently, 4T1 cells were plated in a 12-well plate with incubation for 12 h and then were cultured with lentiviruses (multiplicity of infection (MOI) was 10) and polybrene (10 μg mL^−1^) for 24 h. Then, the transfected 4T1 (4T1^CD47^) cells were screened out with dihydropenicillin and were detected by CLSM and flow cytometry. Besides, the proteins of 4T1 and 4T1^CD47^ cells were obtained from RIPA buffer and loaded with 10% SDS polyacrylamide gel, which were then transferred to PVDF membrane and sealed with BSA for 2 h. Finally, the membranes were treated with anti-CD47 and anti-GAPDH and then were imaged by a chemiluminescence analyzer. To obtain 4T1 cell membrane with high CD47 expression, 4T1^CD47^ cells were resuspended with precooled 0.25 × PBS (phosphate buffer saline) containing PMSF, then the above cell solution was repeated freezing and thawing for three times and centrifuged at a speed of 3000 rpm min^−1^ (10 min) at 4 °C, the supernatant was further centrifuged (13,500 rpm min^−1^, 30 min) at 4 °C. Finally, 4T1^CD47^ cell membrane fragments were extruded 12 times by an Avanti mini extruder.

#### *Synthesis of Ti*_*3*_*C*_*2*_* Nanosheets*

The Ti_3_C_2_ nanosheets were obtained by a sonication exfoliation. In brief, bulk Ti_3_C_2_ were added into deionized water at the concentration of 2 mg mL^−1^, and then, the dispersion was treated with an ultrasonic probe (1200 W) for 15 h, followed by ultrasonic cleaning (300 W) for 12 h. Then, the final dispersion was centrifuged at 7000 rpm min^−1^ for 10 min to remove large Ti_3_C_2_, and the suspension was further centrifuged (13,000 rpm min^−1^) for 30 min to obtain Ti_3_C_2_ nanosheets.

#### *Synthesis of TGCT (Ti*_*3*_*C*_*2*_*-GOX-CPO/TPZ)*

To obtain TGC, GOX (2 mg), CPO (50 U) and Ti_3_C_2_ nanosheets (2 mg) were dispersed into PBS (2 mL) with magnetic stirring for 24 h. During the time, EDC (20 mg) and NHS (30 mg) were joined to active Ti_3_C_2_ nanosheets. At last, the obtained mixture was centrifuged (13,000 rpm min^−1^, 10 min) and rinsed with PBS to obtain TGC. TG and TC were obtained by the same above method. To obtain TGCT, TPZ (0.5, 1, 2, 4 or 8 mg) was dissolved into 2 mL of deionized water containing 2 mg of TGC (Ti_3_C_2_-GOX-CPO) under vigorous stirring. Then, TGCT was obtained by centrifugation at the speed of 13,000 rpm min^−1^, the drug loading efficiency and entrapment efficiency were calculated by UV–Vis at 470 nm.

To obtain m_e_TGCT, the TGCT was mixed with equal amounts of the above obtained cell membrane, and the mixture was extruded for 12 passes by Avanti mini extruder (200 nm membrane). Finally, obtained m_e_TGCT was characterized by SDS-PAGE protein analysis. Specifically, m_e_TGCT was dissolved in RIPA lysis buffer with an ice bath for 30 min and prepared in SDS loading buffer. Then, we heated the samples loading buffer to 99 °C. Next, the samples were loaded each well of 10% SDS polyacrylamide gel, and then, the samples were run at the voltage of 80 V (0.5 h) and 120 V (1.5 h), the resulting polyacrylamide gel was stained by Coomassie blue and washed overnight.

The release of TPZ from m_e_TGCT solution (1 mg mL^−1^) was detected in dialysis bag (MWCO = 3000 Da) and placed into 20 mL of PBS with shaking at 37 °C. Then, 0.5 mL of sample solution was got from dialysis bag and the same volume of PBS was added, the release content of TPZ was detected by UV–Vis at 470 nm. Besides, the TPZ release was detected under 808 nm laser irradiation with or without the addition of glucose (4 mg mL^−1^) and Cl^−^ (25 mM) when the sample was exposed to 808 nm laser (1.5 W cm^−2^, 3 min) at 2, 8, and 24 h, respectively.

### Loading Amount and Cascade Catalytic Activity of GOX and CPO

The loading amount of GOX and CPO was detected by BCA protein assay Kit. In brief, GOX (2 mg), CPO (50 U) and Ti_3_C_2_ nanosheets (2 mg) were dispersed into 2 mL of PBS, and then EDC (20 mg) and NHS (30 mg) were added and stirred overnight. TGC was acquired by centrifugal separation, and the supernatant was analyzed to calculate the amount of residual enzyme. The calculation of GOX and CPO loading efficiency (LE) is confirmed as follows:

$$LE(\% ) = \frac{{\left( {m_{i} - m_{r} } \right) \times 100}}{{m_{i} }}$$

where *m*_*i*_ and *m*_*r*_ stand for the initial enzyme mass and the residual enzyme mass, respectively.

To evaluate the cascade catalytic activity of GOX and CPO, glucose (4 mg mL^−1^) was added into the m_e_TGCT (50 μg mL^−1^) solution, and then, the changes of dissolved O_2_ level and the pH were detected.

HClO detection was performed by HClO detection Kit (Amplite fluorescence method). Briefly, different samples were dispersed in PBS solution with glucose (4 mg mL^−1^) and Cl^−^ (25 mM), and laser irradiation groups were treated with 808 nm (1.5 W cm^−2^, 3 min) and 635 nm (0.5 W cm^−2^, 5 min) lasers, and then, the supernatants were collected and treated with HClO detection Kit, the HClO content was monitored according to the OD value of 550 nm. Besides, m_e_TGCT of different concentrations were dispersed in PBS solution containing glucose (4 mg mL^−1^) and Cl^−^ (25 mM) and were treated with HClO detection Kit, the HClO content was monitored according to the OD value of 550 nm.

### Detection of Singlet Oxygen In vitro

The production efficiency of ^1^O_2_ in m_e_TGCT was detected by the absorbance change of the DPBF. Typically, Ti_3_C_2_, m_e_T (CD47-engineering membrane@Ti_3_C_2_) and m_e_TGCT were added into DPBF ethanol solution containing 4 mg mL^−1^ of glucose and 20 mM of NaCl, respectively. Then, the mixture was placed in darkness and exposed to 635 nm (0.5 W cm^−2^) laser. The DPBF absorption at 420 nm was detected at different points of time. Besides, ^1^O_2_ quantum yields (*Φ*Δ) were also detected via DPBF indicator, and the O_2_-saturated solution of Ti_3_C_2_, m_e_T, m_e_TGCT and MB with DPBF (20 μg mL^−1^) was exposed to 635 nm laser (0.5 W cm^−2^) for 3 min; the absorbance change of DPBF at 420 nm was recorded every 30 s by the UV–Vis system. Finally, the ΦΔ values of all samples were calculated by the standard of MB (*Φ*Δ = 0.52) [36].

### Detection of the Photothermal Effect In vitro

808 nm laser was used as the NIR light source to evaluate the photothermal effects of m_e_TGCT. Various concentrations of m_e_TGCT (1.5 mL) were added into transparent quartz plates under 808 nm laser (1.5 W cm^−2^) exposure, and the temperature was recorded every 30 s. The photothermal conversion efficiency (*η*) was measured by detecting the temperature change of m_e_TGCT solution under 808 nm laser (1.5 W cm^−2^).

Besides, the photothermal stability of m_e_TGCT was examined by the 808 nm laser, m_e_TGCT solution was exposed (5 min) and then cooled to inceptive temperature naturally with repetition (4 times). In order to investigate the impact of the physiological environment on the stability of m_e_TGCT, which was dispersed in PBS and kept at 37 °C, the particle size and PDI were recorded at the various time by dynamic light scattering (DLS).

### Detection of Cellular Uptake

The cell internalization and subcellular distribution of m_e_TGC were assessed with C6 fluorescent dye via flow cytometry and CLSM, respectively. At a word, 4T1 cells were cultured for 12 h and subsequently cultured with TGC/C6 (50 μg mL^−1^) and m_e_TGC/C6 (50 μg mL^−1^), and then, 4T1 cells were collected for flow cytometry detection and stained by DAPI (10 μg mL^−1^) for CLSM.

### m_e_TGC Promoted M1 Macrophages Phagocytosis of Tumor Cells

To detect the impact of m_e_TGC on the M1 macrophage phagocytosis of 4T1 cells, 1000 ng mL^−1^ of LPS was used to polarize RAW264.7 cells into M1 macrophages, which were cultured with m_w_TGC (wild-type cell membrane@Ti_3_C_2_-GOX-CPO/TPZ) and m_e_TGC for 2 h, respectively. After that, mCherry-labeled 4T1 cells were collected and added into per well at the quantity ratio of 1:5 (macrophages/cancer cells). After 4 h incubation, M1 macrophages were analyzed via CLSM and flow cytometry.

### Cellular ROS and Thermal Imaging Evaluation

The ROS generation of m_e_TGCT was confirmed using DCFH-DA. In short, 4T1 cells were plated for 24 h and subsequently cultured with m_e_TGCT for 4 h, and then DCFH-DA was added into the different samples containing Ti_3_C_2_, m_e_T (m_e_@Ti_3_C_2_), m_e_TG, m_e_TC, m_e_TGC and m_e_TGCT (50 μg mL^−1^) for ROS detection. With or without 635 nm laser (0.5 W cm^−2^, 5 min) treatment, 4T1 cells were analyzed via flow cytometry and CLSM.

### Intracellular Hypoxia Detection In vitro

The intracellular hypoxia status was evaluated by Hypoxyprobe™-1 plus kit. The composition of hypoxyprobe-1 is a pimonidazole hydrochloride, which could be reduced by the intracellular nitroreductase to produce the stable adducts with thiol (sulphydryl) groups in the tissue, and then FITC-marked antibody (FITC-Mab1) could bind to these adducts to reflect the cellular O_2_ level by immunofluorescence method. In brief, 4T1 cells were plated for 12 h of incubation, and then, the medium was replaced with m_e_T, m_e_TG, m_e_TC and m_e_TGC (50 μg mL^−1^). After 4 h incubation, laser groups were treated with 635 nm laser (0.5 W cm^−2^, 5 min), and then, pimonidazole HCl was used to pre-treat the cells for 1 h and stained using FITC-Mab1 for another 30 min. Finally, the cell hypoxia status was analyzed via CLSM and flow cytometry.

### 2.10. Cytotoxicity Evaluation in vitro

The cytotoxicity was measured via MTT method. 4T1 cells were cultured for 12 h, and then, m_e_T, m_e_TG, m_e_TC, m_e_TGC, TGCT, and m_e_TGCT with concentration of 50 μg mL^−1^ were used to treat with 4T1 cells for 4 h, which were irradiated with 808 nm (1.5 W cm^−2^, 3 min) and 635 nm (0.5 W cm^−2^, 5 min) lasers and continued to be cultured to 24 h. MTT (5 mg mL^−1^) was added for a further 4 h incubation, the absorption value was recorded at 570 nm after the addition of DMSO.

Besides, the efficacy of every formulation was evaluated by a live/dead cell experiment. Briefly, 4T1 cells were processed with different formulations (50 μg mL^−1^ of m_e_T, m_e_TG, m_e_TC, m_e_TGC, and m_e_TGCT) for 24 h, and the laser irradiation conditions were the same as the MTT assay. Finally, the cells were stained by Calcein-AM/PI and recorded by CLSM.

Finally, the anti-proliferation capacities of m_e_TGCT were further measured by cell apoptosis experiments. Firstly, 4T1 cells were cultured with m_e_T, m_e_TG, m_e_TC, m_e_TGC, and m_e_TGCT at the same concentration (50 μg mL^−1^) for 24 h, and laser irradiation condition was the same as the MTT assay, and then, the cells were treated with Annexin V-FITC/PI and detected by flow cytometry subsequently.

### 2.11. Tumor Targeting Capacity of m_e_TGC

All animal experiments were approved in agreement with the Animal Ethics Committee and Use of Sun Yat-sen University (2,021,000,656). BALB/c female mice (3–5 weeks old) were given 4T1 cells (1 × 10^6^) by subcutaneous injection to create the animal model, and then, the mice were intravenously injected with Ti_3_C_2_/Cy5.5, m_w_TGC/Cy5.5 or m_e_TGC/Cy5.5 (2 mg kg^−1^) when the tumor volume reached to 100 mm^3^. The fluorescence images were monitored (excitation: 670 nm, emission: 710 nm) at different time. Lastly, the tumors and major organs were dissected and monitored after 24 and 120 h administration.

### 2.12 Antitumor Evaluation in vivo

BALB/c female mice were given 4T1 cells by subcutaneous injection to create the animal model. After fed for 7 days, the mice (n = 5 per group) were intravenously injected with different samples at a dosage 2 mg kg^−1^ as follows: PBS, m_e_T (CD47-engineering membrane@Ti_3_C_2_), m_e_TG (CD47-engineering membrane@Ti_3_C_2_-GOx), TGC (Ti_3_C_2_-GOX-CPO), m_e_TGC (CD47-engineering membrane@Ti_3_C_2_-GOX-CPO), TGCT (Ti_3_C_2_-GOX-CPO/TPZ), m_e_TGCT (CD47-engineering membrane@Ti_3_C_2_-GOX-CPO/TPZ), TGCT+laser, m_e_TCGT+laser, at the first day and fourth day, respectively. Then, tumors of the laser groups were irradiated by 808 nm (1.5 W cm^−2^, 2 min) and 635 nm (0.5 W cm^−2^, 5 min) lasers at 12 h post-injection. And the tumor volume and body weight were measured regularly during a month. Afterward, the major organs were peeled for hematoxylin and eosin (H&E) and terminal transferase dUTP (TUNEL) detection, and the tumor tissues were weighed and photographed. The tumor volume was calculated via the formula: V = length × (width)^2^/2, and V/V_0_ in each group was used to normalize tumor volume, V_0_ is the inceptive tumor volume. Furthermore, obtained serum was used for evaluating the alanine aminotransferase (ALT), aspartate aminotransferase (AST), albumin (ALB), alkaline phosphatase (ALP), blood urea nitrogen (BUN), creatinine (CR), γ-glutamyltransferase (γ-GT), total protein (TP) and glucose (GLU) values. In addition, the tumors were stained FITC-Mab1 by immunofluorescence staining to assess the deoxy-generation ability of m_e_TGCT in vivo.

### Statistical Analysis

All data were used in the manuscript as mean ± standard deviation. Statistical analysis was constructed by the two-tailed Student’s t test.

## Results and Discussion

### Characterization of 4T1 Cell Membrane with High-expressed CD47

**Fig. 1:**
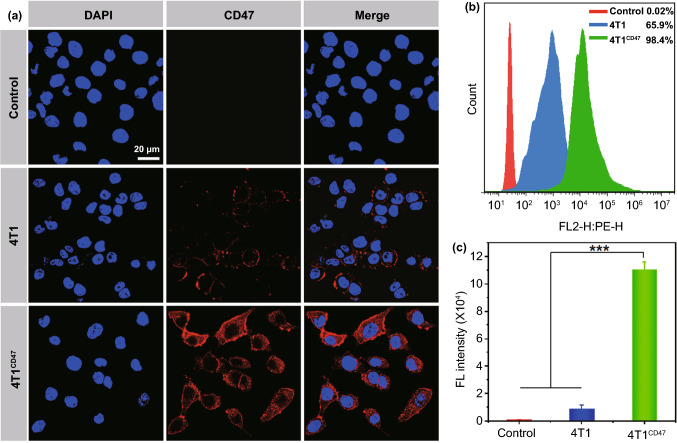
**a** CLSM images and **b** flow histogram of the level of CD47 expression. **c** Quantitative analysis of the CD47 expression. (***p < 0.001, *n* = 3)

To obtain 4T1 cell membrane with high-expressed CD47, we firstly transfected 4T1 cells with lentiviruses vectors to prepare gene-engineering 4T1 cells with overexpression of CD47, and the transfected 4T1 cells (4T1^CD47^) were detected using anti-CD47/PE staining by CLSM and flow analysis. As illustrated in Fig. 1a, the non-transfected 4T1 cell group showed slightly red fluorescence signal, while 4T1^CD47^ cells displayed the strongest fluorescence signal intensity, verifying the successful transfection of 4T1 cells with high CD47 expression. Quantitatively, the percentage of CD47/PE positive cells in 4T1^CD47^ cells group reached 98.4%, which was dramatically as high as that in 4T1 cells group (Fig. 1b), and the fluorescence intensity of 4T1^CD47^ cells was 12.8 times as vigorous as that of 4T1 cells by quantitative analysis (Fig. 1c). Besides, it was also identified the high expression of CD47 on 4T1^CD47^ cells by western blot analysis (Fig. S1). These results all suggested the successful preparation of 4T1 cells with high-CD47 expression.

### Characterization of m_e_TGCT (CD47-engineering Membrane @Ti_3_C_2_-GOX-CPO/TPZ)

**Fig. 2:**
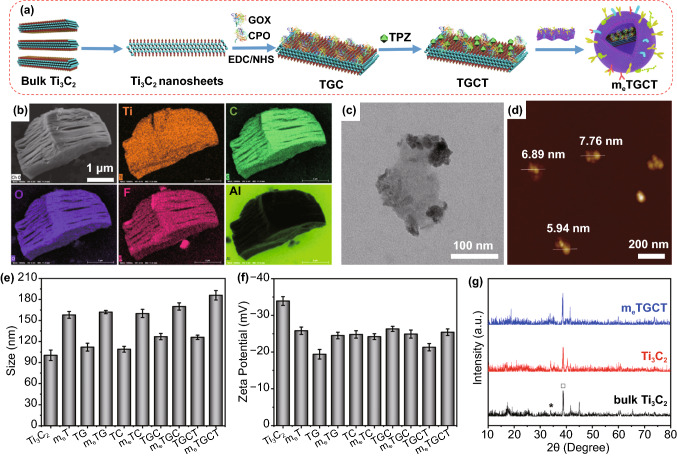
**a** Design principle of the synthesis of m_e_TGCT. **b** SEM and element mapping of the bulk Ti_3_C_2_. **c** TEM image of Ti_3_C_2_ nanosheets. **d** AFM image of the exfoliated Ti_3_C_2_ nanosheets. **e** The size and **f** apparent zeta charge of Ti_3_C_2_, m_e_T, TG, m_e_TG, TC, m_e_TC, TGC, m_e_TGC, TGCT, m_e_TGCT. **g** XRD images of the bulk Ti_3_C_2_, Ti_3_C_2_ nanosheets and m_e_TGCT

The cascaded-enzyme nanoreactor (m_e_TGCT) was synthesized by conjugating GOX and CPO onto the surface of Ti_3_C_2_ nanosheets loading with TPZ and further encapsulated with 4T1 cell membrane with high-CD47 expression (Fig. 2a). Ti_3_C_2_ nanosheets were prepared from the bulk layer-structured Ti_3_C_2_ by ultrasonication. Scanning electron microscopy (SEM) images showed the accordion-like layer structures of origin Ti_3_C_2_, and the element mapping of the bulk Ti_3_C_2_ sample confirmed the absence of the Al element and the existence of O and F elements (Fig. 2b). The morphology of obtained Ti_3_C_2_ nanosheets was characterized by TEM (Fig. 2c) and SEM (Fig. S2a) images, revealing the ultrathin and transparent flake with the size of 100 nm and exhibiting the typical 2D topology. The atomic force microscopy (AFM) image suggested that the thickness of the as-synthesized Ti_3_C_2_ nanosheets was ∼6 nm (Fig. 2d). The exfoliated Ti_3_C_2_ nanosheets also performed a size of 115 nm (Fig. 2e) with a charge of -28.9 mV (Fig. 2f). The infrared spectroscopy (Fig. S2b) and Raman spectroscopy (Fig. S2c) confirmed that the surface of as-exfoliated Ti_3_C_2_ nanosheets was rich in –COOH and –OH groups. Subsequently, GOX and CPO were conjugated onto Ti_3_C_2_ nanosheets with amido bonds, and the loading efficiency (LE) was detected to be 15.69%. For comparison, the GOX LE was 20.79% for Ti_3_C_2_-GOX (TG) and CPO LE was 9.35% for Ti_3_C_2_-CPO (TC), respectively (Fig. S3). After loading, the particle size of Ti_3_C_2_ nanosheets (115 nm) increased to ~ 145 nm (Fig. 2e), and the zeta potential ( − 28.9 mV) slightly changed to -24.5 mV (Fig. 2f), implying the successful loading of GOX and CPO. Afterward, hypoxia-activated prodrug TPZ was loaded onto Ti_3_C_2_-GOX-CPO (TGC) through physical interactions. The ultraviolet–visible (UV–Vis) spectra demonstrated the successful loading of TPZ in the TGC (Fig. S4a), when the ratio of TGC/TPZ was 1:2, the loading efficiency (LE) and entrapment efficiency (EE) were 63.06% and 85.12% (Fig. S4b), respectively. The zeta potential of Ti_3_C_2_-GOX-CPO/TPZ (TGCT) increased to − 18.5 mV (Fig. 2f). To obtain m_e_TGCT, TGCT was embedded with 4T1 cell membrane with CD47 high expression by repeated extrusion. As shown in Fig. S5, m_e_TGCT exhibited a transverse dimension (160 nm) and transparent membrane coating, confirming the successful packaging of 4T1 cell membrane. What’s more, the size of m_e_TGCT widened to 180 nm (Fig. 2e), and the zeta charge swelled to ~ -14.6 mV (Fig. 2f). In addition, the protein expression of m_e_TGCT was detected by SDS-PAGE identification, and 4T1 cell membrane proteins were largely retained, and the bands of GOX (80 kDa) and CPO (46 kDa) were also observed in the m_e_TGCT group (Fig. S6), confirming that GOX and CPO were successfully accommodated in m_e_TGCT. These results all verified the successful preparation of m_e_TGCT. Of special note, the hydrodynamic size and PDI of m_e_TGCT were kept constant for at least 7 days, and the good dispersion of m_e_TGCT in PBS was observed after 7 days in contrast to the precipitation occurring in TGCT, confirming the excellent stability of m_e_TGCT in the physiological condition (Fig. S7). Moreover, almost no visible difference was observed in X-ray diffraction (XRD) patterns among m_e_TGCT, Ti_3_C_2_ nanosheets and the bulk Ti_3_C_2_ (Fig. 2g), indicating that m_e_TGCT reserved the uniform crystal structure and crystallinity as Ti_3_C_2_.

### Detection of Cascade Catalytic Reactions and Drug Release

**Fig. 3:**
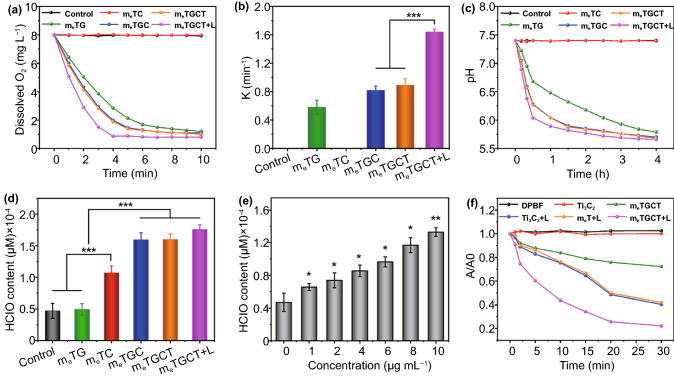
**a** Changes of dissolved O_2_ in PBS containing various samples with/without 635 nm (0.5 W cm^−2^) and 808 nm (1.5 W cm^−2^) lasers under 4 mg mL^−1^ of glucose and 25 mM of Cl^−^. **b** O_2_ consumption rates based on **a**. **c** Changes of pH in PBS containing different samples. **d** HClO production content of PBS containing different samples. **e** HClO production content of PBS containing m_e_TGCT with different concentrations in the presence of 4 mg mL^−1^ of glucose and 25 mM of Cl^−^, *p* values were contrast with the group (concentration: 0 μg mL^−1^). **f** A/A0 of the DPBF treated with Ti_3_C_2_, m_e_T and m_e_TGCT with or without 635 nm light irradiation. (***p < 0.001, **p < 0.01, *p < 0.05, n = 3)

It has been reported that chemical modifications could reduce the catalytic capabilities of enzymes [37]. Thus, the catalytic activities of GOX and CPO in m_e_TGCT were evaluated. GOX catalyzes glucose to generate H_2_O_2_ and gluconic acid under O_2_, leading to pH decrease and O_2_ consumption [38–40], and then, CPO could catalyze H_2_O_2_ and Cl^−^ to produce HClO. On the basis of these, the cascaded-enzyme catalytic capacities of m_e_TGCT were measured by O_2_ consumption, pH decrease and HClO generation. Firstly, the dissolved O_2_ level of various groups in PBS containing 4 mg mL^−1^ of glucose and 25 mM of Cl^−^ were measured by an O_2_ probe. As displayed in Fig. 3a, m_e_TC group exhibited similar results as the control group, indicating that m_e_TC did not consume O_2_. However, with the addition of GOX, the dissolved O_2_ level quickly declined and gradually reached a platform. The O_2_ level of m_e_TG gradually decreased from 8.1 mg L^−1^ to a balanceable value of 1.4 mg L^−1^ within 10 min, and the consumption rate of O_2_ obviously increased in m_e_TGC and m_e_TGCT groups, indicating that the presence of CPO accelerated GOX-catalyzed reaction activities. Importantly, under the 808 + 635 nm lasers irradiation, the O_2_ level in m_e_TGCT group decreased faster and reached 0.8 mg L^−1^ within 4 min, which might be because that 635 nm laser irradiation could induce the energy transfer of Ti_3_C_2_ that reacts with O_2_ to produce ^1^O_2_, and the increased temperature caused by 808 nm laser could enhance the enzymatic activities of GOX and CPO. Besides, the O_2_ consumption rate curves in Fig. 3a accorded well with the exponential equation (y = a × e^−kx^ + b), where *x* and *y* were the reaction time and O_2_ concentration, respectively. The constant *k* interpreted as the O_2_ consumption rate is shown in Fig. 3b, demonstrating m_e_TGCT could rapidly consume O_2_ with laser irradiation. Considering that the temperature enhancement by laser irradiation might accelerate enzymatic catalytic rate, the photothermal effect of m_e_TGCT was also evaluated via 808 nm laser. As shown in Fig. S8a, m_e_TGCT displayed a photothermal effect with concentration and time dependence, the temperature of m_e_TGCT (25 μg mL^−1^) rapidly added up to 18 °C under 808 nm laser irradiation for 10 min, exhibiting a superior photothermal capability, while temperature of solution without m_e_TGCT rose mildly (ΔT≈ 1.7 °C). The photothermal stability is further measured and shown in Fig. S8b, and there was no distinct variation in photothermal capacity of m_e_TGCT during four heating cycles, presenting excellent photothermal stability. Besides, the photothermal conversion efficiency of m_e_TGCT was computed as 53.87% based on the cooling curve (Fig. S8c), which is remarkably higher than most photothermal agents (PTAs) such as black phosphorus (BP) nanoparticles (29.47%), graphene oxide (25%), MoS_2_ nanosheets (27.6%), PEG modified antimonene quantum dots (PEG-AMQDs, 45.5%) [41, 42]. Therefore, the results proved that m_e_TGCT possessed good photothermal conversion properties, which would benefit for improving the enzyme activity. Next, we further evaluated the enzymatic activity of m_e_TGCT by pH change in PBS containing 4 mg mL^−1^ of glucose and 25 mM of Cl^−^, which was monitored by a pH meter. As clarified in Fig. S9, pH values of PBS (containing 4 mg mL^−1^ of glucose) remained constant at 7.4 without GOX treatment. With the increase in concentration of additional GOX, the pH of glucose solution gradually declined and dropped to 4.24 when the concentration of GOX was 50 μg mL^−1^, verifying that GOX could improve glucose oxygenolysis in the manner of time and concentration dependence. In addition, the pH values of glucose solution with different treatments were detected to evaluate the catalytic activity of m_e_TGCT (Fig. 3c). The pH values of the control and m_e_TC groups remained constant, while the m_e_TG group exhibited certain pH changes, illustrating the catalytic activities of m_e_TG were retained. Compared with m_e_TG group, m_e_TGC and m_e_TGCT groups exhibited an enhanced drop of pH, demonstrating that CPO could accelerate the GOX-catalyzed reaction process, while the significantly reduced pH values were shown in the m_e_TGCT + L group, which was ascribed to the enhanced catalytic reactivity by hyperthermia induced by laser irradiation.

The cascade catalytic activity of m_e_TGCT was further evaluated by CPO-catalyzed product. CPO could catalyze H_2_O_2_ and Cl^−^ into highly cytotoxic HClO, so we detected the generated HClO of the m_e_TG, m_e_TC, m_e_TGC, m_e_TGCT, and m_e_TGCT + L in PBS with the addition of glucose (4 mg mL^−1^) and Cl^−^ (25 mM) to imitate physiological environment. As revealed in Fig. 3d, m_e_TGC group exhibited enhanced HClO generation capacity compared with the m_e_TG and m_e_TC groups, demonstrating the successful cascaded catalysis of GOX and CPO enzymes. The absence of either GOX or CPO could restrict the catalytic activity and limit the generation of HClO. After laser irradiation, the HClO generation of the m_e_TGCT group further increased, which was attributed to the increase in temperature by laser irradiation. Meanwhile, the HClO generation content of m_e_TGCT performed a concentration-dependent manner (Fig. 3e). To further explore the HClO generation capacity of the CPO catalysis, we evaluated the HClO content in the m_e_TGCT group with additional H_2_O_2_, glucose or Cl^−^ in PBS. As shown in Fig. S10, the generated HClO content gradually increased with the incremental concentration of H_2_O_2_, glucose or Cl^−^, demonstrating the significant impacts of H_2_O_2_, glucose and Cl^−^ for the cascaded catalysis. Afterward, the relative enzyme activities of GOX, CPO in different samples with or without laser irradiation are assessed in Fig. S11, and m_e_TGC exhibited increased enzymatic activity of GOX and CPO compared to m_e_TG and m_e_TC due to the cascaded-enzyme reaction, and m_e_TGCT with laser irradiation further improved the enzyme activity of both GOX and CPO, which could be ascribed to the rise of temperature. The above results indicated that the cascaded-enzyme nanoreactor possessed excellent enzyme catalytic capacity.

Finally, the ^1^O_2_ generation capacity of m_e_TGCT was evaluated by DPBF indicator. As shown in Fig. 3f, compared with DPBF alone group, the rapid downswing of absorbance ratio at 420 nm was observed in Ti_3_C_2_ + L and m_e_T + L solution within 30 min, indicating the excellent ^1^O_2_ generation capacity of Ti_3_C_2_, and cell membrane coating did not influence the ROS generation capacity of Ti_3_C_2_. A sharper downtrend was observed in m_e_TGCT + L solution owing to the combination of the cascade enzymatic catalysis and photodynamic effect, and the ^1^O_2_ quantum yield of m_e_TGCT increased from 0.18 without laser irradiation to 0.60 with laser irradiation (Fig. S12). Therefore, it could be concluded that m_e_TGCT possessed a significantly superior capacity of ^1^O_2_ generation, which is promising for tumor treatment studies in vitro and in vivo.

The TPZ release is determined in Fig. S13. Compared with TPZ-releasing percentage of 26.1% under normal conditions, 808 nm laser irradiation enhanced the TPZ release due to the heat effect produced by Ti_3_C_2_. Especially, nearly of 65.9% of TPZ was released from m_e_TGCT under the existence of glucose and Cl^−^, it could be deduced that m_e_TGCT catalyzed the glucose to generate gluconic acid, which decreased the electrostatic interaction between TPZ and Ti_3_C_2_, thus accelerating the drug release [43, 44].

### Cellular Uptake Study of m_e_TGC

**Fig. 4:**
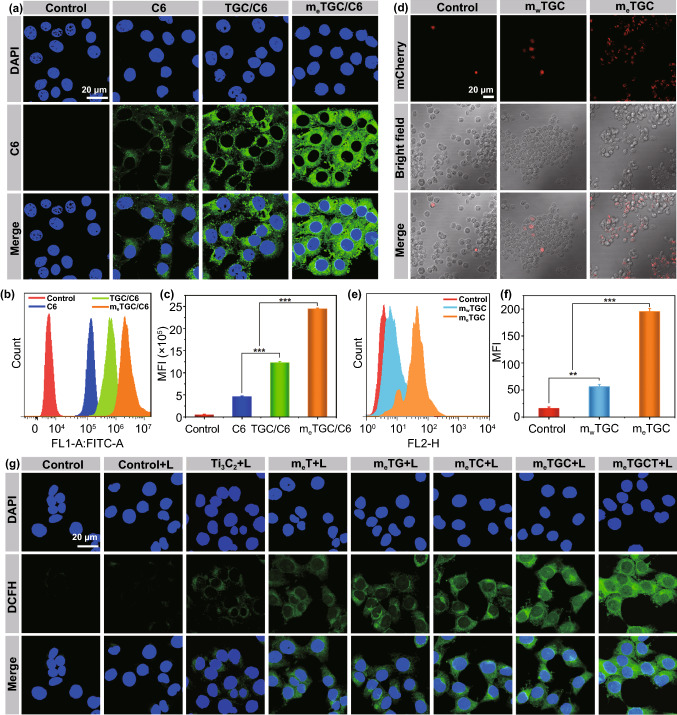
**a** CLSM results of 4T1 cells uptake toward TGC/C6 and m_e_TGC/C6 at 4 h. **b** Flow cytometric results and **c** quantitative flow analysis of 4T1 cells uptake of C6-labeled different formulations. **d** CLSM results of 4T1 cells were swallowed by macrophages after different treatments with m_w_TGC and m_e_TGC. **e** Flow histogram analysis and **f** quantitative flow analysis of 4T1 cells swallowed by macrophages after treatments with m_w_TGC and m_e_TGC. **g** CLSM results of intracellular ROS level. (***p < 0.001, **p < 0.01, *n* = 3)

It had been reported that cancer cell membrane-coated nanocarriers exhibited preferential homologous tumor targeting capacity [45–49]. Thus, the homologous targeting capacity of m_e_TGC on 4T1 cells was measured via flow cytometry and CLSM, and C6 dye was defined as a fluorescence probe to label m_e_TGC. As illustrated in Fig. S14, only tiny amounts of C6 in cells were observed after 10 h incubation with the pure C6, the mean fluorescence intensities of TGC/C6 enhanced over time and achieved the maximum at 8 h. By comparison, m_e_TGC/C6 group exhibited stronger fluorescence intensities at all times, indicating the well homologous targeting ability. Besides, the intracellular locations of TGC and m_e_TGC against 4T1 cells were detected by CLSM (Fig. 4a–-c), the signal (green) of the C6-labeled cascaded-enzyme nanoreactor surrounding the nuclei in the m_e_TGC/C6 group was strongest among all groups, and the quantitative flow cytometry analysis showed the fluorescence intensity of m_e_TGC/C6 group was 1.9 times as high as that of TGC/C6 group (Fig. 4c), further indicating cell membrane coating could improve tumor cell uptake for the cascaded-enzyme nanoreactor.

### m_e_TGC Promoted M1 Macrophages Phagocytosis of Cancer Cells

CD47, overexpressed on the surface of m_e_TGC, could interact with SIRPα presented on macrophages and protected m_e_TGC from the phagocytosis by macrophages through sending “don’t eat me” signal. At the same time, CD47-overexpressed m_e_TGC would block the interaction between macrophages and tumor cells through competitively occupying SIRPα, which could enhance the macrophage-mediated phagocytosis for tumor cells [33, 34]. Therefore, we firstly constructed cellular uptake experiments to confirm the immune escape capacity of the m_e_TGC by flow cytometry. As shown in Fig. S15, an obviously decreased fluorescence intensity of cells treated with m_e_TGC/C6 was found as compared to the TGC/C6 group, and the cell uptake of TGC/C6 was three times as strong as that of m_e_TGC/C6 group, indicating that coating TGC with CD47 high-expressed 4T1 cell membrane could effectively inhibit the immune recognition to achieve immune escape. To evaluate whether m_e_TGC could induce M1 macrophages-mediated 4T1 cells phagocytosis, we pretreated M1 macrophages with m_e_TGC for 2 h and then explored the co-culture of M1 macrophages and mCherry-labeled 4T1 cells to investigate the phagocytosis effect of macrophages by CLSM and flow cytometry. As clarified in Fig. 4d, the red signal (mCherry-labeled tumor cells) in the m_e_TGC group was obviously stronger than that of in control and m_w_TGC (wild-type cell membrane-coated TGC) groups, indicating that high-expressed CD47 on the surface of the m_e_TGC could promote macrophages-mediated 4T1 cells phagocytosis, which was because that CD47 on m_e_TGCT could competitively combine with SIRPα, thus increasing the interaction between macrophages and 4T1 cells [50–53]. Similar results were confirmed in flow cytometry analysis (Fig. 4e, f), the fluorescence signal in m_e_TGC group was three times as strong as that in m_w_TGC group, further clarifying the m_e_TGC could preoccupy SIRPα, leading to the saturation of SIRPα, then enhancing macrophage-mediated phagocytosis of tumor cells [35].

### Cellular ROS Detection

The intracellular ROS level was also measured on 4T1 cells after treatment with m_e_TGCT by flow cytometer and CLSM. As revealed in Fig. 4g, the fluorescence signal was nearly detected in the control group regardless of 635 nm light irradiation, and that of the Ti_3_C_2_ group slightly enhanced, indicating the ROS generation capacity of Ti_3_C_2_ under laser irradiation. The fluorescence signal in m_e_T group was as strong as that of Ti_3_C_2_ treatment, which could be ascribed to the increased uptake by tumor cells owing to homologous targeting mediated by the cell membrane (Fig. 4a–-c). Further enhanced DCF fluorescence was found in the m_e_TG and m_e_TC groups, which was because m_e_TG could catalyze glucose and O_2_ into H_2_O_2_, and m_e_TC could catalyze endogenous H_2_O_2_ into HClO, and m_e_TGC group exhibited more intensive fluorescence signal than the m_e_TG and m_e_TC groups, indicating that the cascaded catalytic reaction could effectively generate ROS. The fluorescence intensities of the m_e_TGCT group were further strengthened, which might be because that the secondary electrons derived from the irradiated Ti_3_C_2_ could be captured by TPZ to further generate hydroxyl radicals by a reductive reaction [54]. Most importantly, the strongest DCF signal was observed in m_e_TGCT + L group, suggesting m_e_TGCT could effectively generate ROS in living cells under 635 nm light. The results of the flow cytometry also verified that m_e_TGCT + L group exhibited the strongest ROS production among all the treatment groups, which was almost 13-fold higher than that of m_e_T group (Fig. S16). These results confirmed that m_e_TGCT could generate sufficient ROS via several different mechanisms, thereby achieving promising overall therapeutic effects in vitro.

### Intracellular Hypoxia Detection of 4T1 Cells

**Fig. 5:**
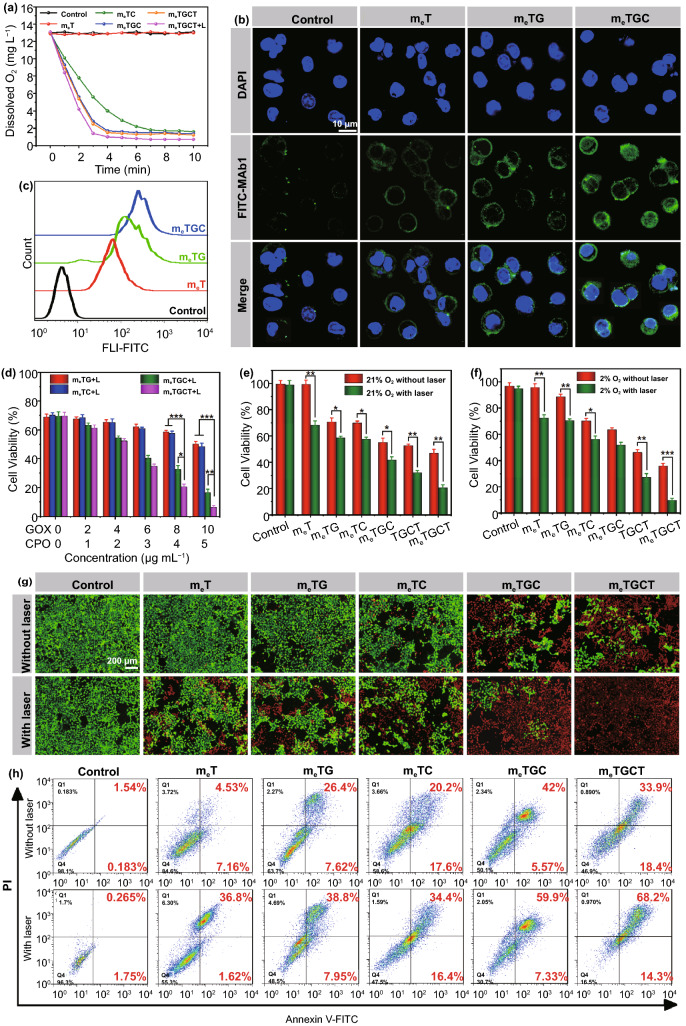
**a** Change of dissolved O_2_ in 4T1 cells treated with different formulations. **b** CLSM images and **c** flow histogram of cellular hypoxia of 4T1 cells incubated with different formulations using a hypoxia probe. **d** Cell viability of 4T1 cells treated with m_e_TG, m_e_TC, m_e_TGC, and m_e_TGCT with various GOX/CPO concentrations under 808 + 635 nm lasers irradiation. Cell viability of 4T1 cells incubated with various formulations in **e** 21% O_2_ condition and **f** 2% O_2_ condition with/without laser irradiation. **g** Live/dead staining images and **h** cell apoptosis of 4T1 cells after various treatments for 4 h (***p < 0.001, **p < 0.01, *p < 0.05, *n* = 5)

The intracellular O_2_ consumption effects were evaluated by incubating 4T1 cells with different samples and detecting the changes of the dissolved O_2_ in the DMEM medium. As shown in Fig. 5a, a quick depletion of O_2_ was found in the m_e_TGC and m_e_TGCT groups, and the most rapid O_2_ depletion rate was exhibited in the m_e_TGCT group with 635 nm laser irradiation, indicating that the cascaded-enzyme could increase intracellular O_2_ consumption. In addition, the cellular hypoxia level was further assessed by CLSM and flow cytometry using a FITC-Mab1 antibody (Fig. S17). It could find that the fluorescence intensities of all groups with 635 nm light irradiation were significantly higher than those of groups without laser irradiation, attributing to the consumption of O_2_ caused by the photodynamic effect. Besides, the merely slight green fluorescence of hypoxia-positive signal was found in the m_e_T group with 635 nm laser irradiation, attributing to the small amount of O_2_ consumption caused by PDT effect. 4T1 cells treated with m_e_TG exhibited enhanced fluorescence intensity, whereas the strongest fluorescence intensity was observed in the m_e_TGC group, which ascribed to the more O_2_ depletion by cascaded-enzyme (GOX and CPO) and PDT effect of Ti_3_C_2_ (Fig. 5b, c). The aforementioned results indicated that the cascaded-enzyme catalytic reaction could effectively consume cellular O_2_ level for the potential activation of TPZ in treatment studies.

### In vitro Antitumor Effects of m_e_TGCT

Before measuring the antitumor efficiency of cascaded-enzyme nanoreactor, we estimated the real-time temperature variation in cells with 808 nm light irradiation. As clarified in Fig. S18, the temperature in both of experiment groups showed rapid temperature rise, while control group merely rose to 29.6 °C within 3 min of 808 nm laser irradiation. Particularly, the temperature of m_e_TGC group reached to mild hypothermia condition (44.6 °C), which was beneficial to improve the EDT and PDT effects. Afterward, the remarkable capacities of the cascaded catalysis and the therapeutic effects of m_e_TGCT were evaluated by MTT assays in vitro. As displayed in Fig. S19a, b, almost no distinct cytotoxicity of Ti_3_C_2_ and m_e_T indicated their satisfactory biocompatibility. The cell viability decreased with the increment concentration of m_e_T under 808 + 635 nm lasers irradiation, indicating the phototherapy effects of m_e_T. The therapeutic effects of m_e_TG, m_e_TC, m_e_TGC, and m_e_TGCT with various concentrations are shown in Fig. 5d, and m_e_TGCT displayed the highest cytotoxicity against 4T1 cells comparing to other groups, owing to the cancer cell membrane coating prompting more cellular uptake of TGCT, and the cascaded catalytic reaction achieving efficient EDT, as well as hypoxic-activation of TPZ for chemotherapy. Considering that cascaded-enzyme nanoreactor could induce the tumor cell deoxygenation, we evaluated the therapeutic effects of m_e_TGCT in normoxic or in hypoxic environments with/without 808 + 635 nm laser irradiation. Firstly, the cytotoxicity of chemotherapeutic effects on 4T1 cells is evaluated in Fig. S19c, free TPZ exhibited almost no cytotoxicity on 4T1 cells owing to the unactivated status of TPZ in normoxic environment. Besides, Fig. 5e, f shows that cell viability of TPZ free groups (m_e_T, m_e_TG, m_e_TC, and m_e_TGC) in hypoxic environment was similar to that in normoxic environment, while the anticancer effects of the m_e_TGCT and TGCT groups in hypoxic environment were enhanced compared to those in the normoxic environment, which could be attributed to the deoxygenation-activated chemotherapy effect of TPZ [27]. To statistically confirm the combination effects of PTT, PDT, EDT and chemotherapy on 4T1 cells inhibition, the combination index (CI) was calculated on the basis of the Chou-Talalay assay [55]. The 50% inhibition concentration (IC_50_) of TPZ group in hypoxia environment was 26.92 μg mL^−1^, and the IC_50_ of m_e_TGC under laser irradiation group was 51.21 μg mL^−1^. The IC_50_ of m_e_TGCT under laser irradiation and hypoxia environment was 19.27 μg mL^−1^, and the CI was calculated as 0.824, indicating there were synergistic effects between phototherapy and chemotherapy. In addition, we further measured the therapeutic effects of m_e_TGCT in media with high glucose level or low glucose level (Fig. S19d). Compared with the high cell viability of m_e_TG group in high glucose level media, the m_e_TGCT group exhibited the enhanced cell cytotoxicity on account of the cascaded EDT effects. Besides, in low glucose level media, the cell viability of the m_e_TGCT group also reached 12.5% at 24 h under laser irradiation, owing to EDT effect and cell starvation induced by the complete glucose decomposition, indicating the exclusive effect of the m_e_TGCT nanoreactor in tumor cell growth suppression with or without glucose supply. These results strongly indicated that the combined phototherapy/EDT/chemotherapy with m_e_TGCT treatment under laser irradiation could effectively kill tumor cells.

On purpose of evaluating visually anticancer effects of m_e_TGCT in vitro, Calcein-AM/PI staining fluorescence imaging was further conducted to observe the cell live/dead states (Fig. 5g). Briefly, a small number of cells turned into orange or red (dead) in the m_e_TG and m_e_TC groups, indicating m_e_TG and m_e_TC could induce slight cells death. Under laser irradiation, the number of red cells increased, ascribing to the phototherapy effect of Ti_3_C_2_. Specially, almost all cells were stained red in m_e_TGCT group with laser irradiation, which further confirmed the best anticancer efficacy of m_e_TGCT treatment under laser irradiation toward 4T1 cells. In addition, the apoptosis experiment confirmed similar results (Fig. 5h). The proportions of early and late apoptosis cells were 11.69%, 34.02%, 37.8%, and 47.57% after treatment with m_e_T, m_e_TG, m_e_TC, and m_e_TGC, respectively, which increased to 38.42%, 46.75%, 50.8%, and 67.23% after being exposed to laser irradiation, respectively, while that of m_e_TGCT with laser irradiation (82.5%) was far higher than the above groups, demonstrating definite superiority for tumor therapy comparing to single EDT, phototherapy or chemotherapy. Therefore, the combination of tumor phototherapy, EDT and chemotherapy was a safe and effective synergistic strategy for tumor treatment.

### In vivo Fluorescence Imaging

**Fig. 6:**
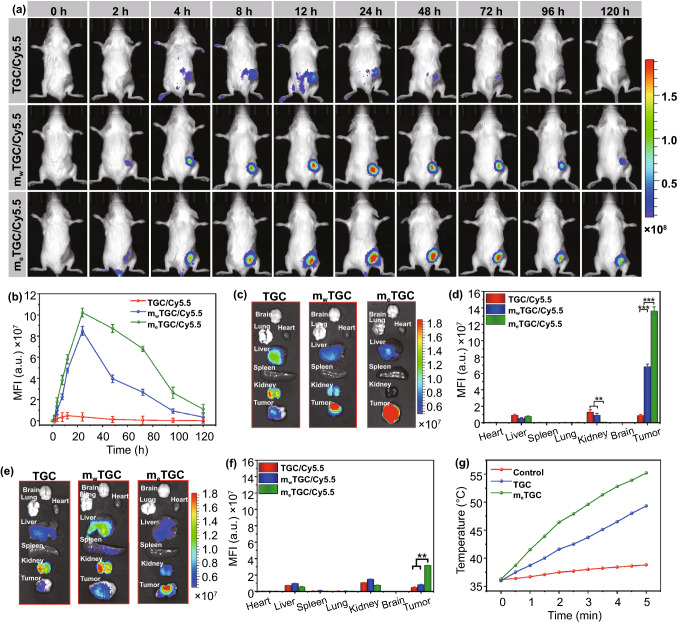
**a** Biodistribution of TGC/Cy5.5, m_w_TGC/Cy5.5, and m_e_TGC/Cy5.5 in 4T1 tumor-bearing mice. **b** Corresponding average fluorescence intensity based on **a**. Fluorescence imaging of organs and tumor **c** at 24 h and **e** at 120 h post-injection. Corresponding average fluorescence intensity of major organs and tumor at 24 h **d** based on **c** and 120 h **f** based on **e**. **g** Temperature changes of tumor region under 808 nm light (1.5 W cm^−2^) within 5 min (***p < 0.001, **p < 0.01, *n* = 3)

Inspired by the excellent capacity of homologous targeting in vitro, the tumor targeting and accumulation performance of m_e_TGCT were determined in vivo. As shown in Fig. 6a, b, the m_e_TGC/Cy5.5 fluorescence intensity increased persistently and achieved a peak at 24 h. Although the fluorescence intensity decreased gradually after 72 h, it was still detected at 120 h, demonstrating the continued retention of m_e_TGC in tumor tissue. m_w_TGC/Cy5.5 group showed similar fluorescence changes within 24 h, while the fluorescence signal decreased quickly after that. The dramatic distinction was attributed to the enhanced immune escape and long circulation effect of m_e_TGC due to high-expressed CD47. In contrast, the fluorescence signal of TGC/Cy5.5 was weaker at the tumor site and faded away quickly after 12 h post-injection. Moreover, the fluorescence intensity of m_e_TGC/Cy5.5 was always 7 to 34 times as high as that of TGC/Cy5.5 at different time intervals, which clearly exhibited the long-term tumor duration property. Besides, all tumors and organs were obtained after 24 or 120 h post-injection (Fig. 6c, e), semiquantitative analysis of the fluorescence intensity is shown in Fig. 6d, f. In the TGC/Cy5.5 group, Cy5.5 mainly accumulated in liver and kidney after 24 h, while that of m_e_TGC/Cy5.5 group was obviously observed in tumor, 15.6-fold as high as that of TGC/Cy5.5 group, exhibiting the enhanced tumor accumulation and prolonged blood circulation after coating with the homologous tumor cell membrane with high CD47 expression. Such long circulation time would effectively enhance the therapeutic efficiency of nanoreactor. Most importantly, this highly specific tumor recognition capacity of m_e_TGC could significantly strengthen the therapeutic effects, as well as reduce the side effects.

### 3.10. In vivo Photothermal Imaging and Tumor Hypoxia Detection

To evaluate the in vivo photothermal effect, the temperature changes of the tumor region were detected (Figs. 6g and S20). In control group, the temperature showed negligible change within 2 min, while that in m_e_TGC treated group rapidly rose from 36.3 to 46.4 °C within 2 min, which could provide a mild hypothermia condition for the enhancement of EDT and PDT effects.

After proving the tumor accumulation of m_e_TGCT, we explored the intratumoral hypoxia status after 48 h intravenous administration of m_e_TGCT in vivo. As displayed in Fig. S21, there was almost no fluorescence signal of hypoxia (green) in PBS and m_e_T groups, while the increased fluorescence intensity in the treatment groups with GOX indicated the aggravation of tumor hypoxia status due to O_2_ consumption after GOX catalyzing the glucose and O_2_. Besides, the treatment groups with GOX and CPO showed the greatly enhanced fluorescence intensity, which was ascribed to the cascaded EDT effect increasing O_2_ consumption. After laser irradiation, the intratumoral O_2_ level was further exhausted due to PDT and EDT effects, leading to the enhanced tumor hypoxia. Such an O_2_ consumption dynamic in tumor would be conducive to activate chemotherapeutic effect of TPZ for the enhanced antitumor efficiency.

### 3.11. In vivo Antitumor Therapy

**Fig. 7:**
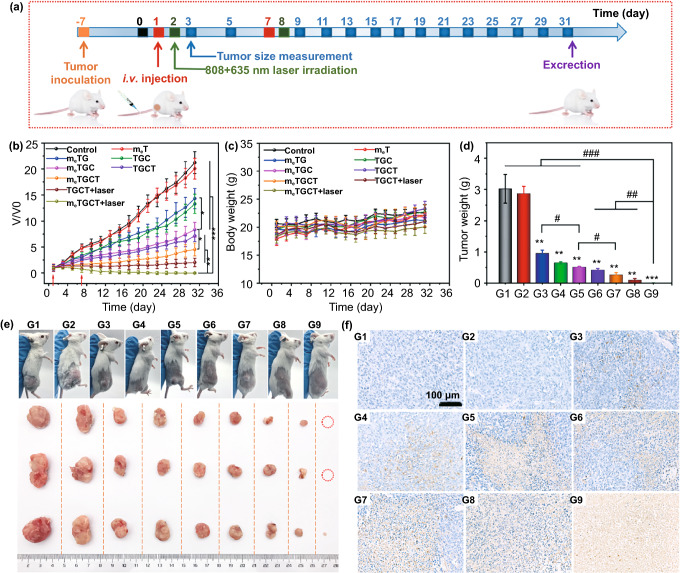
**a** Therapy process of m_e_TGCT. **b** The variation in V/V0 in different groups. **c** Variations in body weight and **d** tumor weight during various treatments. **e** Photographs of mice and tumor tissues on day 31. **f** TUNEL staining of tumors were harvested from the mice of different treatments. (G1) Control, (G2) m_e_T, (G3) m_e_TG, (G4) TGC, (G5) m_e_TGC, (G6) TGCT, (G7) m_e_TGCT, (G8) TGCT+laser, (G9) m_e_TGCT+laser (***p < 0.001, **p < 0.01 and *p < 0.05 as contrast with Control group, ^*###*^*p* < 0.001, ^*##*^*p* < 0.01, ^*#*^*p* < 0.05, *n* = 5)

Finally, 4T1 tumor-bearing me systemic antitumor activity of m_e_TGCT. The therapy process and therapeutic effects of m_e_TGCT are shown in Fig. 7a, b. The tumor sizes in control and m_e_T groups increased quickly, and the slight tumor growth was inhibited by m_e_TG via tumor starvation. The m_e_TGC exhibited moderate tumor growth inhibition with V/V_0_ value of 8.1 on day 31, due to the generation of plentiful HClO caused by cascaded-enzyme reaction. Comparing to TGCT and m_e_TGC groups, the mice treated with m_e_TGCT presented a slow growth of tumors and the final V/V_0_ was 4.5, due to the efficient homologous targeting capacities and starvation/EDT/chemotherapy effects. Besides, under laser irradiation, m_e_TGCT exhibited the strongest therapeutic outcomes (V/V0 = 0.003) among all the groups through the tumor targeting phototherapy and EDT magnified chemotherapy, indicating that the biomimetic cascaded-enzyme nanoreactor offered an optimal strategy for tumor treatment.

Similarly, the weight (Fig. 7d) and photographs (Fig. 7e) of tumors harvested from the mice on day 31 further attested the superior tumor suppression effect of m_e_TGCT with laser irradiation. It was noted that there were two mice treated with m_e_TGCT and laser irradiation got completely tumor remission at the end of the treatment. Meanwhile, compared to the control group where all mice died, the mice of m_e_TGCT + laser treatment all lived for 31 days and there were two mice got complete elimination of tumor until they were dissected, demonstrating this nanoreactor could extend the survival period of tumor-bearing mice (Fig. S22). And the body weights of the mice showed no distinct variations within 31 days (Fig. 7c), indicating the low systemic toxicity of m_e_TGCT.

In addition, the major physiological and biochemical parameters in serum of the mice after treatments were also evaluated. As illustrated in Fig. S23, liver function (ALT, AST, ALB, ALP, TP, γ-GT), kidney function indexes (BUN, CR) and GLU level were in the normal range after treatment with m_e_TGCT, confirming the biosafety of the cascaded-enzyme nanoreactor. Furthermore, H&E staining analysis of the main organs also demonstrated the biosafety of m_e_TGCT during the treatment (Fig. S24). The results indicated that m_e_TGCT could significantly eliminate the tumor with satisfying biosecurity.

To further evaluate the antitumor effects of m_e_TGCT, we dissected the tumors of all groups for terminal transferase dUTP (TUNEL) apoptosis staining (Fig. 7f), H&E and Ki67 antigen results (Figs. S25 and S26), which all suggested that treatment with m_e_TGCT under laser irradiation caused the most significant cell damage and apoptosis in all groups. The superiority of m_e_TGCT treatment further demonstrated the advantages of combined therapy on the basis of cascaded-enzyme nanoreactor-mediated tumor phototherapy, enzyme dynamic therapy and the subsequent deoxygenation-activated chemotherapy.

## Conclusions

Summarily, an intelligent bionic cascaded-enzyme nanoreactor (m_e_TGCT) was performed to achieve amplified antitumor efficiency via phototherapy/enzyme dynamic therapy/chemotherapy. In vitro and in vivo results exhibited that m_e_TGCT nanoreactor was stable during long blood circulation and was able to achieve immune escape and homologous targeting capacities. Besides, the cascaded-enzyme nanoreactor could readily consume glucose and molecular O_2_ at tumor sites under laser irradiation to achieve tumor phototherapy/enzyme dynamic therapy and then created a more hypoxic TME to motivate chemotherapy. Significantly, this bionic cascaded-enzyme nanoreactor provided an innovative insight for the development of complementary patterns for efficient antitumor therapy.

## Supplementary Information

Below is the link to the electronic supplementary material.

Supplementary file1 (PDF 2451 KB)
