# Supplementary material for: A MXene-Based Bionic Cascaded-Enzyme Nanoreactor for Tumor Phototherapy/Enzyme Dynamic Therapy and Hypoxia-Activated Chemotherapy
Source: Nanomicro Lett. 2021 Dec 9;14:22. doi: 10.1007/s40820-021-00761-w (PMC8660948; doi:10.1007/s40820-021-00761-w)
Supplement: Supplementary file 1 — Supplementary file1 (PDF 2451 KB) [file 40820_2021_761_MOESM1_ESM.pdf]

Supporting Information for

A MXene-Based Bionic Cascaded-Enzyme Nanoreactor for Tumor Phototherapy/Enzyme Dynamic Therapy and Hypoxia Activated Chemotherapy

Xiaoge Zhang[1], Lili Cheng[1], Yao Lu[1], Junjie Tang[1], Qijun Lv[2], Xiaomei Chen[1], You Chen[1], Jie Liu[1,*]

[1] School of Biomedical Engineering, Shenzhen Campus of Sun Yat-sen University, No. 66, Gongchang Road, Guangming District, Shenzhen, Guangdong 518107, P. R. China

[2]Department of General Surgery, The Third Affiliated Hospital of Sun Yat-sen University, Guangzhou, Guangdong, 510006, P. R. China

*Corresponding author. E-mail: liujie56@mail.sysu.edu.cn (Jie Liu)

S1 Western Blot Analysis of CD47 Expression

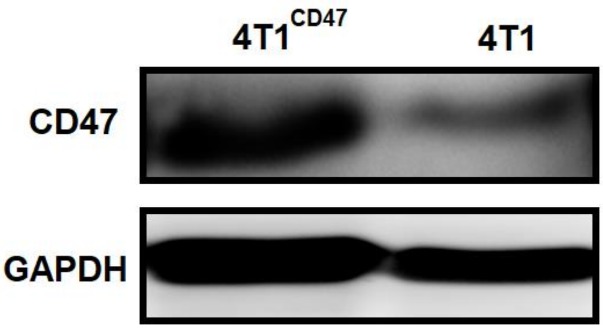

Fig. S1 Western blot analysis of CD47 expression on surface of gene-transfected 4T1 cells ($4T1^{CD47}$) and wild-type 4T1 cells

S2 Characterization of the Exfoliated Ti_3C_2 Nanosheets

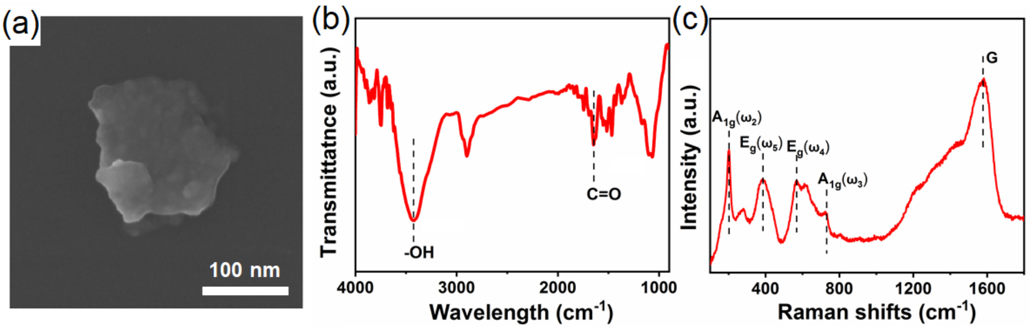

Fig. S2 a SEM image of exfoliated Ti_3C_2 nanosheets. **b** FITR spectra characterization of the exfoliated Ti_3C_2 nanosheets. **c** Raman spectra characterization of the exfoliated Ti_3C_2 nanosheets

S3 Enzyme Loading Efficiency of TGC

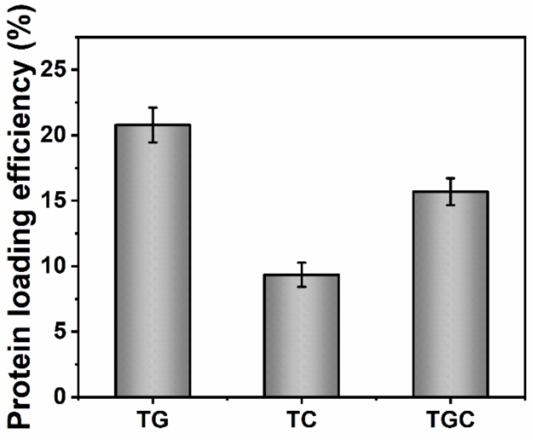

Fig. S3 Protein loading efficiency of TG, TC and TGC

S4 UV Characterization and Drug Loading and Encapsulation Efficiency Detection

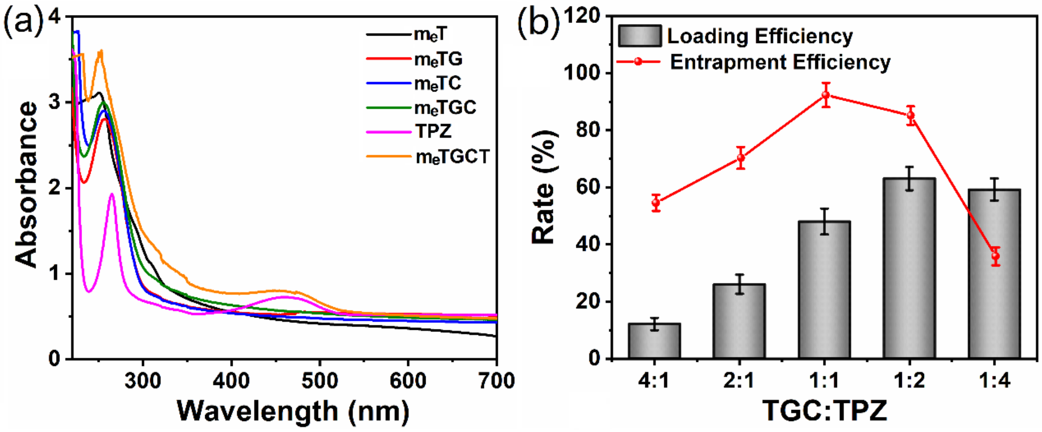

Fig. S4 a UV spectra characterization of different samples. **b** The drug loading and entrapment efficiency of TGC/TPZ with different ratio.

S5 Characterization of the m_eTGCT Nanoreactor

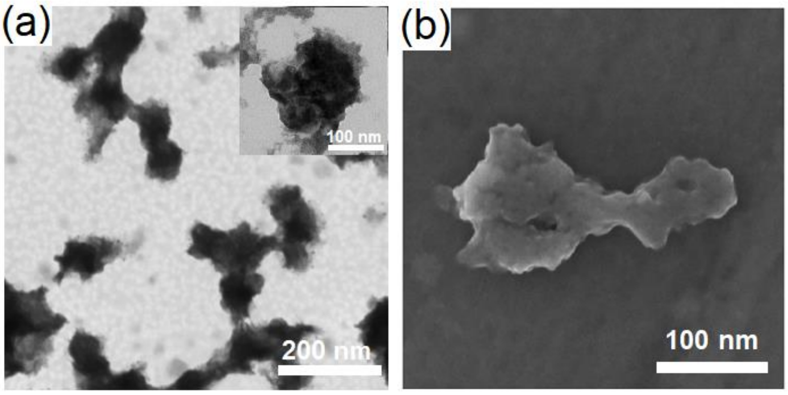

Fig. S5 a TEM image of m_eTGCT. **b** SEM image of m_eTGCT

S6 SDS-PAGE Protein Analysis of m_eTGCT

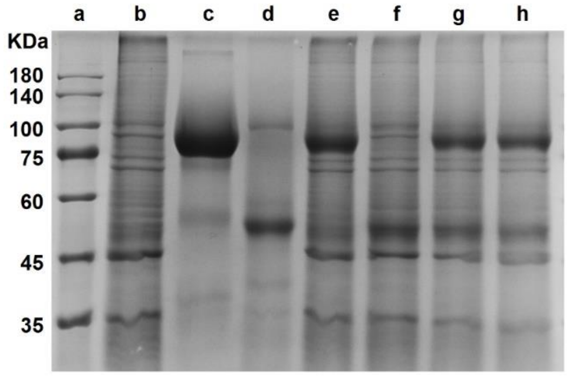

Fig. S6 SDS-PAGE protein analysis of m_eTGCT. **a**) maker, **b**) 4T1^{CD47} cell membrane, **c)** GOX, **d**) CPO, **e**) m_eTG, **f**) m_eTC, **g)** m_eTGC, **h**) m_eTGCT

S7 Stability of m_eTGCT

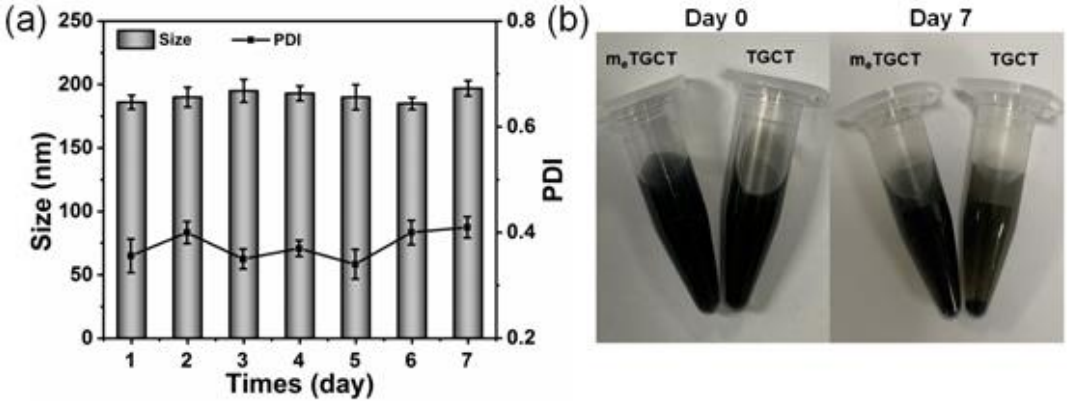

Fig. S7 a The size stability of m_eTGCT in PBS during one week. **b** The photographs of TGCT and m_eTGCT on day 0 and day 7

S8 Photothermal Effects of m_eTGCT *in vitro*

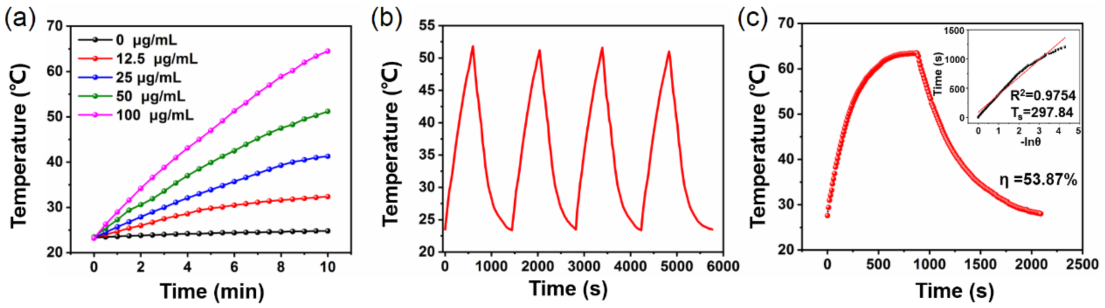

Fig. S8 a Temperature change of m_eTGCT with different concentrations under 808 nm laser (1.5 W cm^{-2}) irradiation for 10 min. **b** Four cycles of temperature variation of m_eTGCT buffer solution (50 μg mL^{-1}) with continuous 808 nm laser irradiation (1.5 W cm^{-2}, 10 min) and natural cooling. **c** The photothermal conversion efficiency of the m_eTGCT solution under 808 nm laser. Inset is plot of linear time data versus –lnθ, which was from the cooling stage

S9 Influence of Different Concentrations of GOX on pH Values of PBS

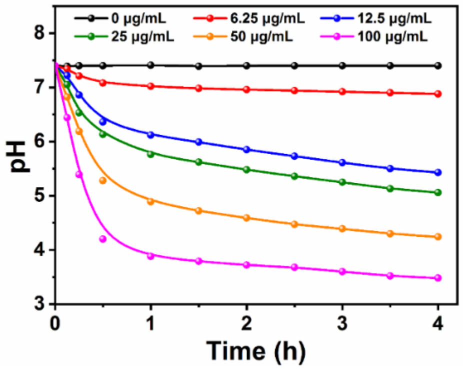

Fig. S9 Time-dependent changes in pH values of PBS containing GOX with different concentrations in the presence of 4 mg mL^{-1} glucose

S10 Detection of the HClO Content

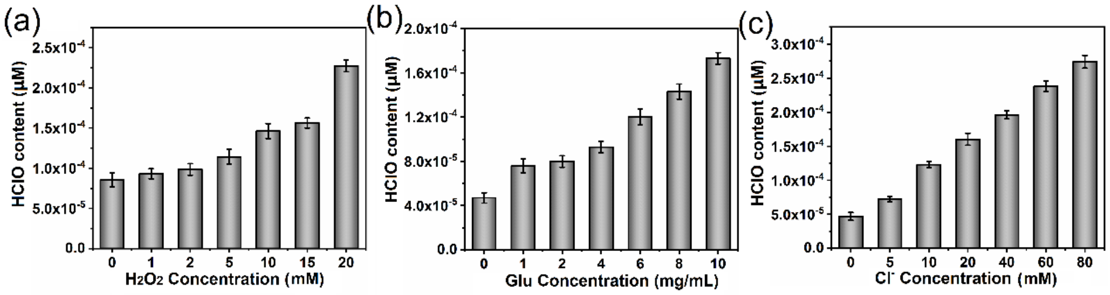

Fig. S10 a HClO production content of PBS containing m$_e$TGCT and different concentrations of H_2O_2 in the presence of 4 mg mL^{-1} glucose and 25 mM Cl$^-$. **b** HClO production content of PBS containing m$_e$TGCT and different concentrations of glucose in the presence of 25 mM Cl$^-$. **c** HClO production content of PBS containing m$_e$TGCT and different concentrations of Cl$^-$ in the presence of 4 mg mL^{-1} glucose

S11 Detection of the Relative Enzymatic Activity

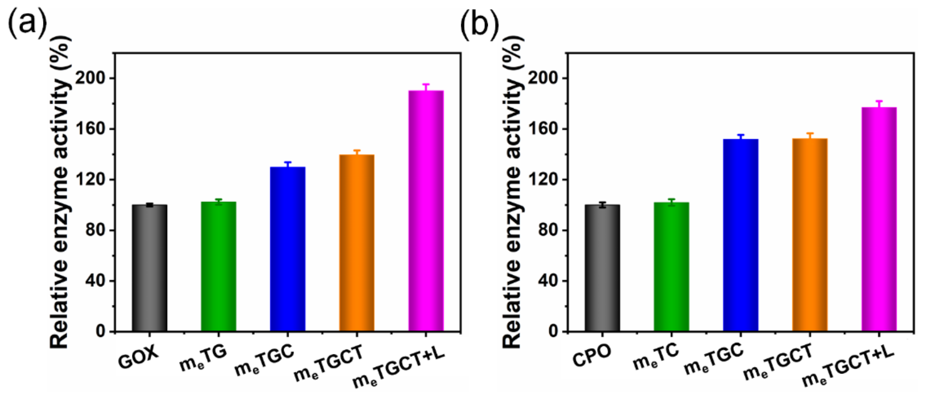

Fig. S11 a The relative GOX enzymatic activity of various samples with or without 808 nm (1.5 W cm^{-2}, 3 min) and 635 nm (0.5 W cm^{-2}, 5 min) lasers irradiation. **b** The relative CPO enzymatic activity of various samples with or without 808 nm (1.5 W cm^{-2}, 3 min) and 635 nm (0.5 W cm^{-2}, 5 min) lasers irradiation

S12 Detection of the $^1O_2$ Quantum Yields

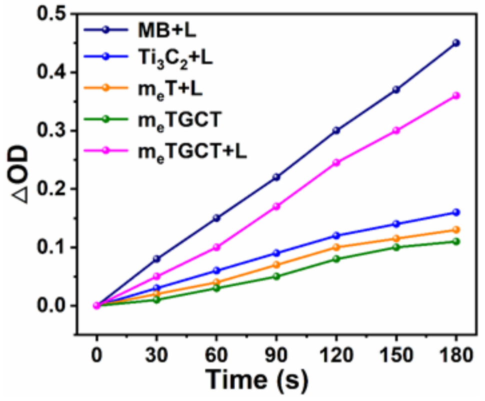

Fig. S12 The time dependent ΔOD of the $^1O_2$ indicator DPBF incubated with Ti_3C_2, m_eT, m_eTGCT and MB with or without 635 nm laser (0.5 W cm^{-2}) for 5 min in O_2-saturated PBS solution

S13 TPZ Release Performances from m_eTGCT

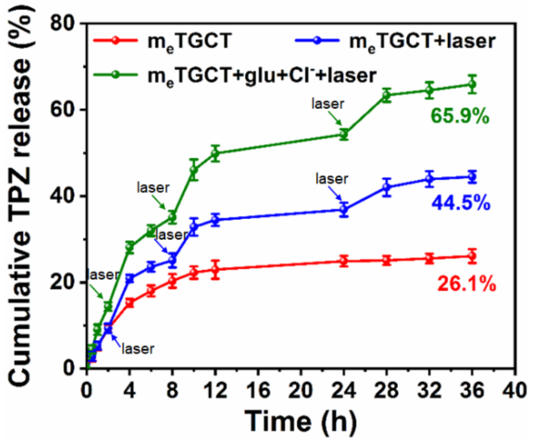

Fig. S13 TPZ release performances from m_eTGCT under different conditions

S14 4T1 Cells Internalization of m_eTGC by Flow Cytometry

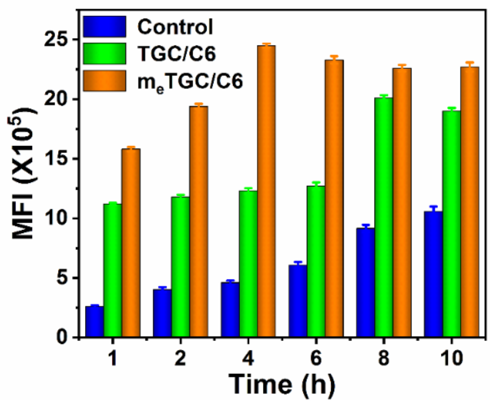

Fig. S14 Flow cytometric results of the uptake amount of C6, TGC/C6, and m_eTGC/C6 by 4T1 cells

S15 Macrophages Internalization of m_eTGC by Flow Cytometry

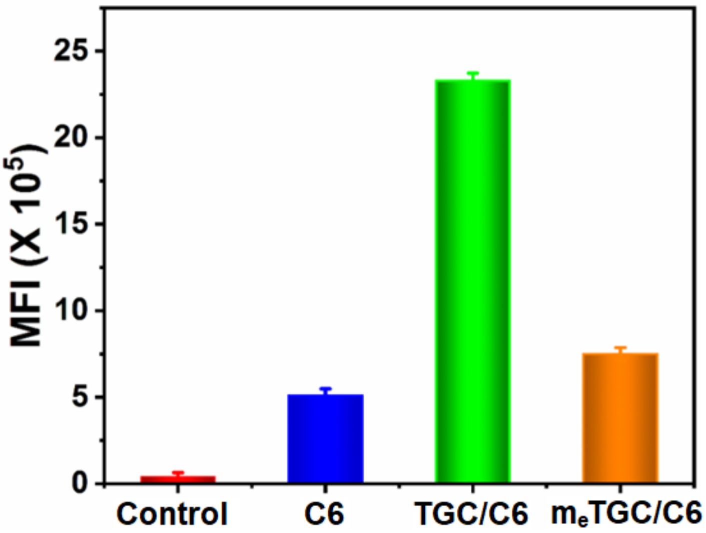

Fig. S15 Flow cytometric results of the uptake amount of C6, TGC/C6 and m_eTGC/C6 by macrophages

S16 Intracellular ROS Detection by Flow Cytometry

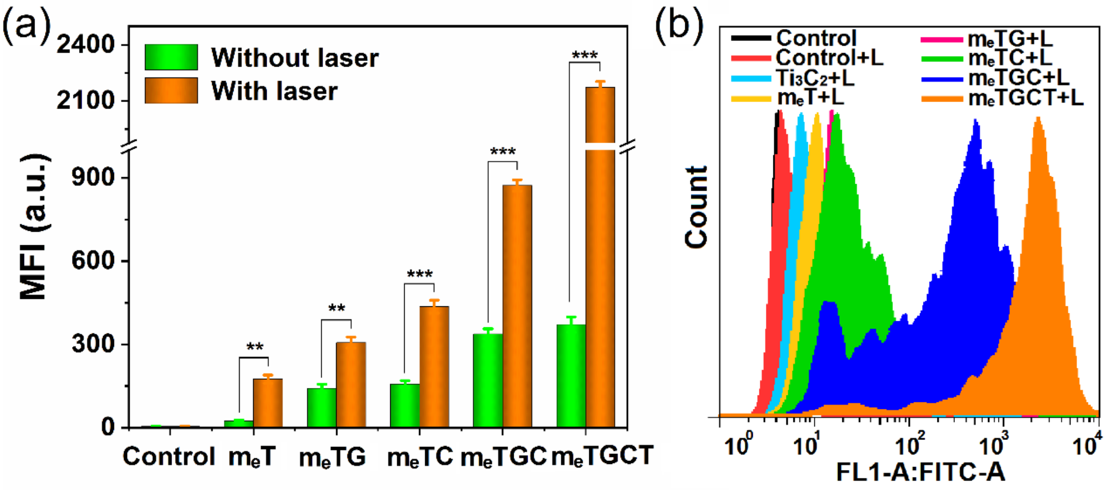

Fig. S16 a Quantitative internalization ROS detection of 4T1 cells with different treatments for 4 h with/without 635 nm laser (0.5 W cm^{-2}, 5 min) by flow cytometry. **b** Intracellular ROS detection in 4T1 cells treated with different samples at 4 h by flow cytometry under 635 nm laser irradiation (0.5 W cm^{-2}, 5 min) (****p* < 0.001, ***p* < 0.01, **p* < 0.05, n = 3)

S17 Cellular Hypoxia Detection of 4T1 Cells

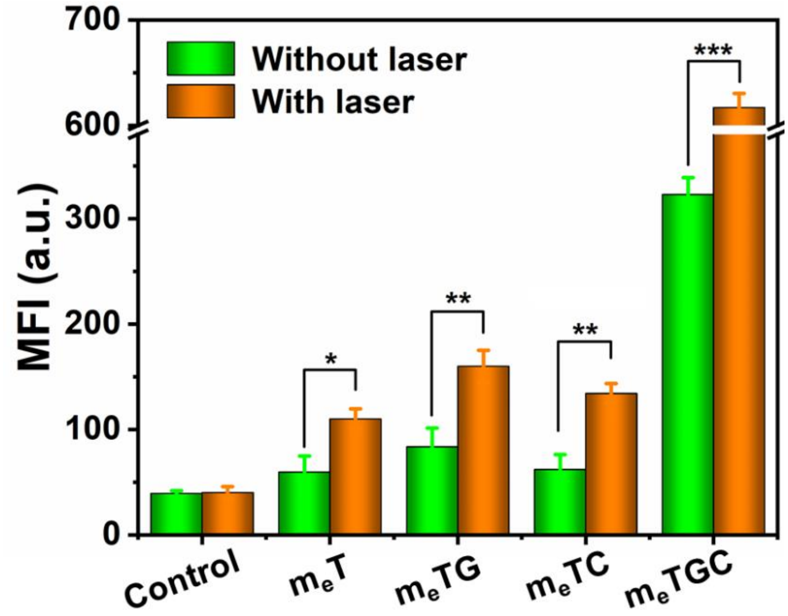

Fig. S17 Cellular hypoxia detection of 4T1 cells incubated with different samples in DMEM by flow cytometry using a hypoxia probe under 635 nm laser irradiation (0.5 W/cm^2) for 5 min. (***$p < 0.001$, **$p < 0.01$, *$p < 0.05$, n = 3)

S18 *In vitro* Photothermal Effects of TGC and m_eTGC

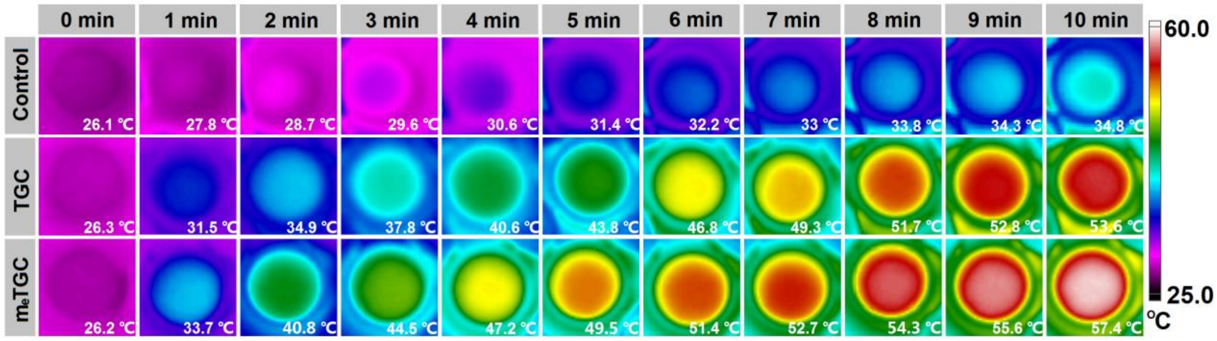

Fig. S18 The temperature changes of 4T1 cells in 96-well plates treated with TGC or m_eTGC for 4 h, before and after 808 nm (1.5 W cm^{-2}) laser irradiation for different time.

S19 *In vitro* Anti-tumor Effects of Ti_3C_2 and m_eT

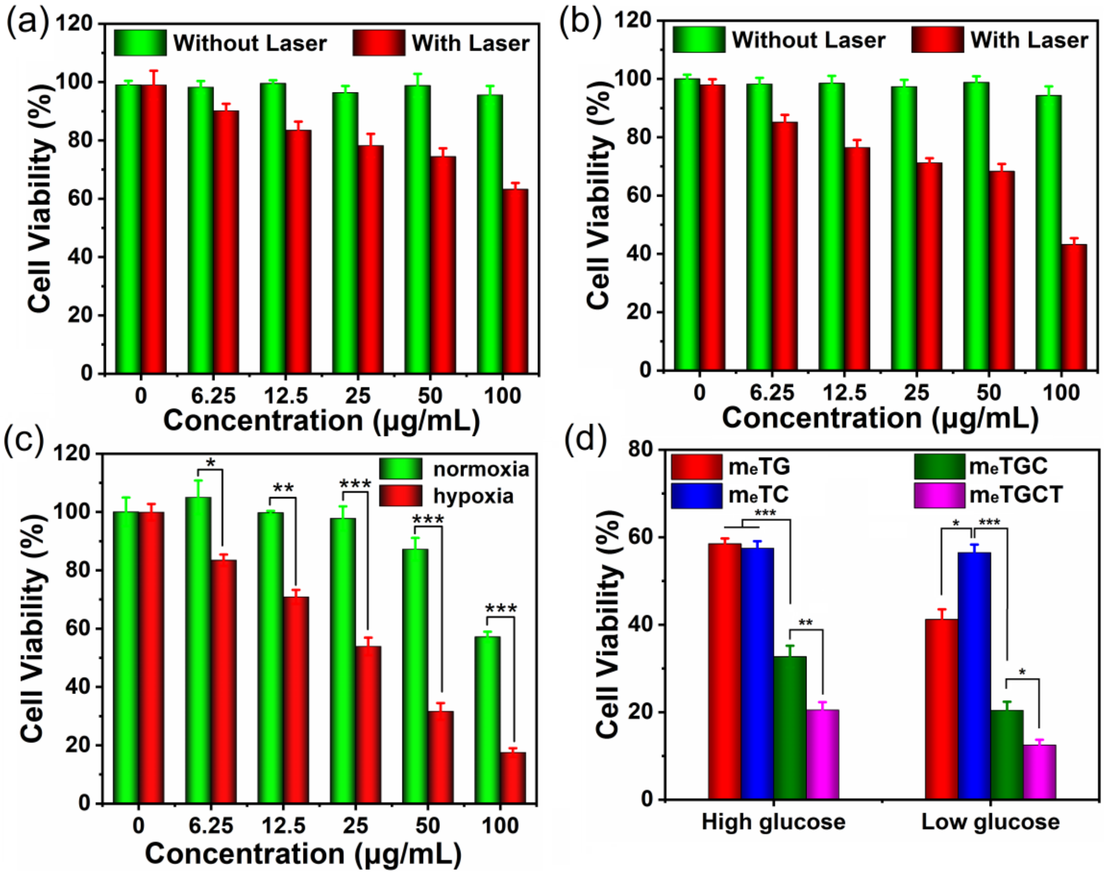

Fig. S19 a 4T1 cell viability of treatment with Ti_3C_2 with or without 808 nm (1.5 W cm^{-2}, 3 min) and 635 nm (0.5 W cm^{-2}, 5 min) laser irradiation. **b** 4T1 cell viability of treatment with m_eT with or without 808 nm (1.5 W cm^{-2}, 3 min) and 635 nm (0.5 W cm^{-2}, 5 min) laser irradiation. **c** 4T1 cell viability of treatment with TPZ of different concentrations in normoxic or hypoxia environment. **d** 4T1 cell viability of varying formulations with high glucose (4.5 mg mL^{-1}) or low glucose (1 mg mL^{-1}) under 808 nm (1.5 W cm^{-2}, 3 min) and 635 nm (0.5 W cm^{-2}, 5 min) laser irradiation. (***$p < 0.001$, **$p < 0.01$, *$p < 0.05$, n = 5)

S20 Photothermal Effect of TGC and m_eTGC *in vivo*

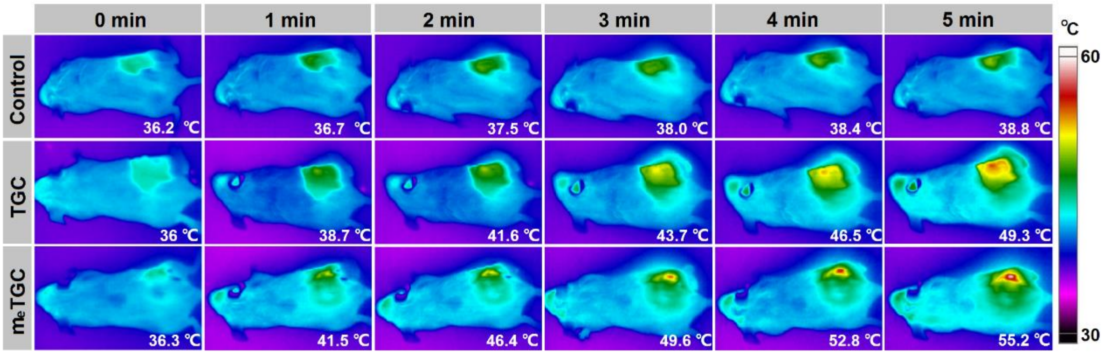

Fig. S20 The temperature images of 4T1 cancer-bearing mice exposed to 808 nm laser (1.5 W cm^{-2}) within 5 min

S21 Representative Immunofluorescence Staining Images of Tumors Stained with the Hypoxyprobe Kit with Different Treatments

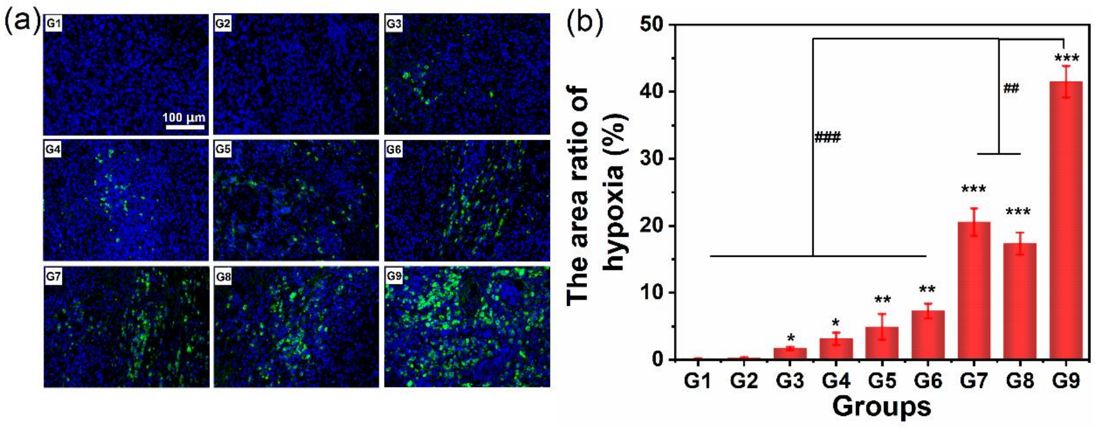

Fig. S21 a Representative immunofluorescence images of tumor slices stained with the hypoxyprobe kit after i.v. injection of G1) Control, G2) m_eT, G3) m_eTG, G4) TGC, G5) m_eTGC, G6) TGCT, G7) m_eTGCT, G8) TGCT+laser, G9) m_eTGCT+laser. **b** The semiquantitative analysis of **a**

S22 Survive Curve of the Mice with Different Treatments

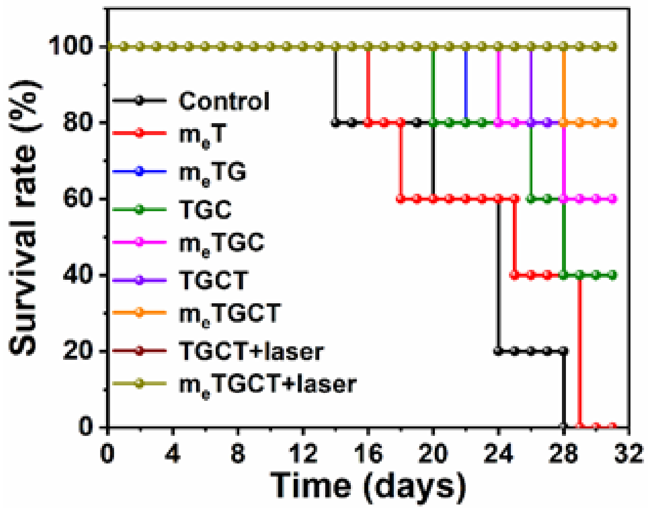

Fig. S22 Kaplan-Meier survival analysis of 4T1 tumor-bearing mice after various treatments

S23 Blood Biochemical Analysis of the Mice with Different Treatments

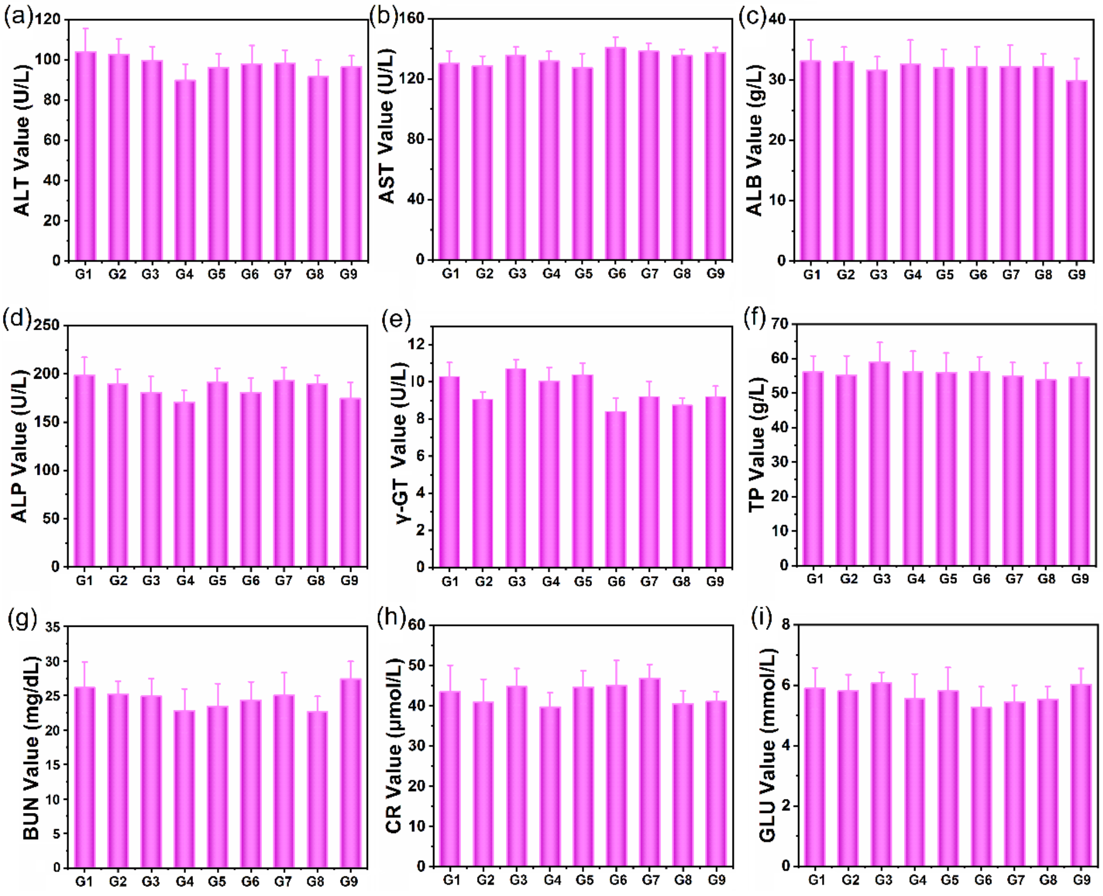

Fig. S23 Blood biochemical results of the mice with different treatments at 21-day post-injection. The results show the mean and SD of **a** alanine aminotransferase (ALT), **b** aspartate aminotransferase (AST), **c** albumin (ALB), **d** alkaline phosphatase (ALP), **e** gamma glutamyl transferase (γ-GT), **f** total protein (TP), **g** blood urea nitrogen (BUN), **h** creatinine (CR) and **i** glucose (GLU). G1) Control, G2) m_eT, G3) m_eTG, G4) TGC, G5) m_eTGC, G6) TGCT, G7) m_eTGCT, G8) TGCT+laser, G9) m_eTGCT+laser

S24 HE Staining of Major Organs

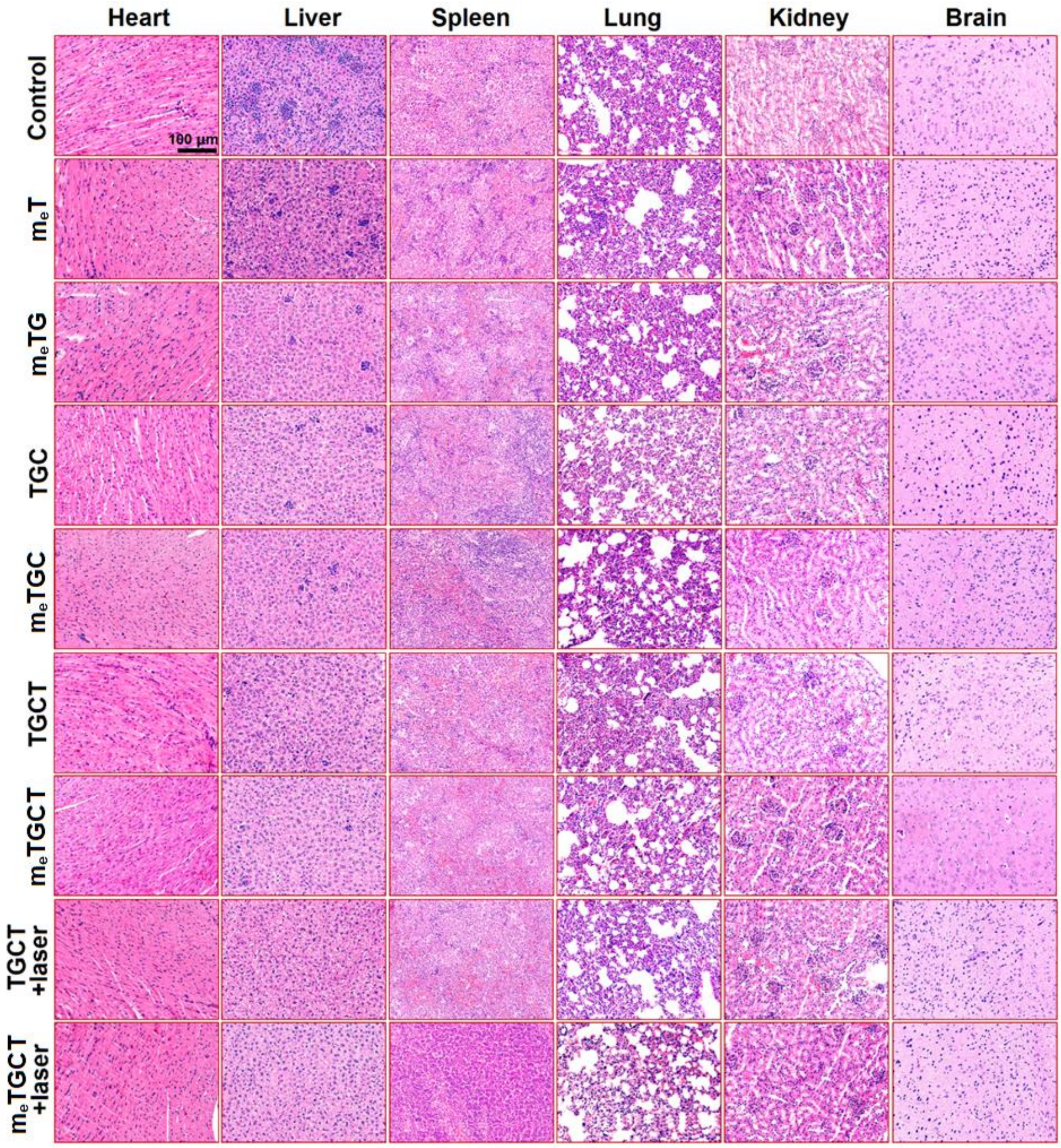

Fig. S24 H&E staining of tissues were harvested from the health mice of different groups

S25 H&E Staining Images of Tumors with Different Treatments

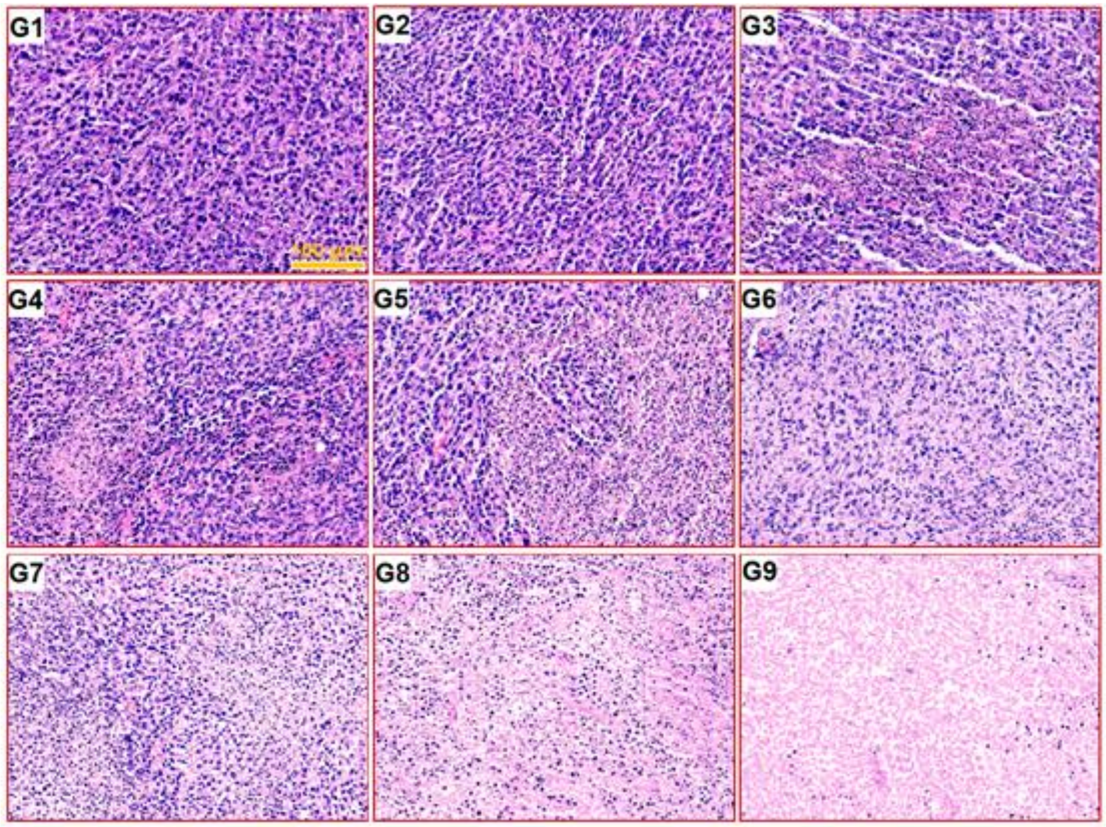

Fig. S25 H&E staining of tumor tissues dissected from the mice in various groups (G1) Control, G2) m_eT, G3) m_eTG, G4) TGC, G5) m_eTGC, G6) TGCT, G7) m_eTGCT, G8) TGCT+laser, G9) m_eTGCT+laser)

S26 Typical Ki67 Staining Images of Tumors with Different Treatments

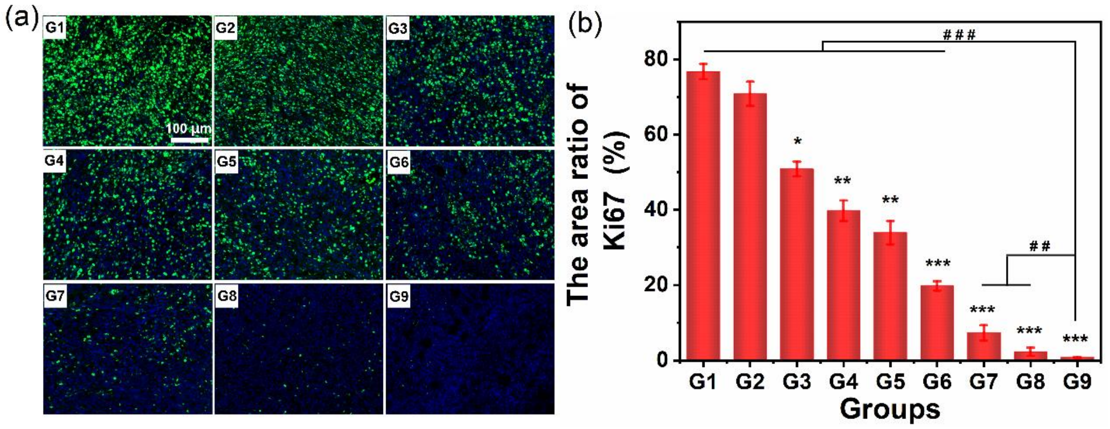

Fig. S26 a Representative immunofluorescence images of proliferating cells (Ki67-positive) of the tumors with different treatments. G1) Control, G2) m_eT, G3) m_eTG, G4) TGC, G5) m_eTGC, G6) TGCT, G7) m_eTGCT, G8) TGCT+laser, G9) m_eTGCT+laser. **b** The semiquantitative analysis of **a**
